# Mesenchymal stromal cells (MSCs) induce *ex vivo* proliferation and erythroid commitment of cord blood haematopoietic stem cells (CB-CD34+ cells)

**DOI:** 10.1371/journal.pone.0172430

**Published:** 2017-02-23

**Authors:** Simone Perucca, Andrea Di Palma, Pier Paolo Piccaluga, Claudia Gemelli, Elisa Zoratti, Giulio Bassi, Edoardo Giacopuzzi, Andrea Lojacono, Giuseppe Borsani, Enrico Tagliafico, Maria Teresa Scupoli, Simona Bernardi, Camilla Zanaglio, Federica Cattina, Valeria Cancelli, Michele Malagola, Mauro Krampera, Mirella Marini, Camillo Almici, Sergio Ferrari, Domenico Russo

**Affiliations:** 1 Unit of Blood Diseases and Stem Cells Transplantation, Department of Clinical and Experimental Sciences, University of Brescia, ASST Spedali Civili di Brescia, Brescia, Italy; 2 Laboratorio CREA (Centro di Ricerca Emato-oncologica AIL), ASST Spedali Civili of Brescia, Brescia, Italy; 3 Department of Experimental, Diagnostic, and Specialty Medicine (DIMES), S. Orsola-Malpighi Hospital, Bologna University School of Medicine, Bologna, Italy; 4 Section of Genomics and Personalized Medicine, Euro-Mediterranean Institute of Science and Technology (IEMEST), Palermo, Italy; 5 Parco Scientifico e Tecnologico Materiali Innovativi e Ricerca Applicata del Mirandolese, Modena, Italy; 6 Applied Research on Cancer-Network (ARC-NET), University of Verona, Verona, Italy; 7 Stem Cell Research Laboratory, Section of Hematology, Department of Medicine, University of Verona, Verona, Italy; 8 Unit of Biology and Genetics, Department of Molecular and Translational Medicine (DMTM), University of Brescia, Brescia, Italy; 9 U.O. of Obstetrics and Gynecology I, ASST Spedali Civili di Brescia, Brescia, Italy; 10 Centro di Ricerche Genomiche, Dipartimento di Scienze della Vita, Università degli Studi di Modena e Reggio Emilia, Modena, Italy; 11 Interdepartmental Laboratory of Medical Research (LURM), University of Verona, Verona, Italy; 12 Department of Transfusion Medicine, Laboratory for Stem Cells Manipulation and Cryopreservation, ASST Spedali Civili di Brescia, Brescia, Italy; 13 Department of Life Sciences, University of Modena and Reggio Emilia, Modena, Italy; Instituto Butantan, BRAZIL

## Abstract

A human bone marrow-derived mesenchymal stromal cell (MSCs) and cord blood-derived CD34+ stem cell co-culture system was set up in order to evaluate the proliferative and differentiative effects induced by MSCs on CD34+ stem cells, and the reciprocal influences on gene expression profiles. After 10 days of co-culture, non-adherent (SN-fraction) and adherent (AD-fraction) CD34+ stem cells were collected and analysed separately. In the presence of MSCs, a significant increase in CD34+ cell number was observed (fold increase = 14.68), mostly in the SN-fraction (fold increase = 13.20). This was combined with a significant increase in CD34+ cell differentiation towards the BFU-E colonies and with a decrease in the CFU-GM. These observations were confirmed by microarray analysis. Through gene set enrichment analysis (GSEA), we noted a significant enrichment in genes involved in heme metabolism (e.g. *LAMP2*, *CLCN3*, *BMP2K*), mitotic spindle formation and proliferation (e.g. *PALLD*, *SOS1*, *CCNA1*) and TGF-beta signalling (e.g. *ID1*) and a down-modulation of genes participating in myeloid and lymphoid differentiation (e.g. *PCGF2*) in the co-cultured CD34+ stem cells. On the other hand, a significant enrichment in genes involved in oxygen-level response (e.g. *TNFAIP3*, *SLC2A3*, *KLF6*) and angiogenesis (e.g. *VEGFA*, *IGF1*, *ID1*) was found in the co-cultured MSCs. Taken together, our results suggest that MSCs can exert a priming effect on CD34+ stem cells, regulating their proliferation and erythroid differentiation. In turn, CD34+ stem cells seem to be able to polarise the BM-niche towards the vascular compartment by modulating molecular pathways related to hypoxia and angiogenesis.

## Introduction

Haematopoiesis is a continuous blood cell production process occurring in the bone marrow niche (BM-niche) through the orchestrated proliferation, self-renewal and differentiation of haematopoietic stem cells (HSCs) [1,2].

The mesenchymal stem/stromal cells (MSCs) play an essential role in the quiescence, proliferation and differentiation of HSCs [3–5]. They are spatially associated with HSCs, adrenergic nerve fibres and arteriolar vasculature and constitute a structurally unique niche made of MSC-HSC pairings, tightly regulated by local input and long-distance cues [6–10].

The CXCL12(SDF-1)/CXCR4 axis is one of the most important biological mechanisms involved in the reciprocal interaction between MSCs and HSCs [11,12]. Experimental data indicate that *in vivo* nestin+ MSC depletion significantly reduces bone marrow homing and haematopoietic progenitor content [13]. In a xenogenic transplantation model, MSCs favour the haematopoietic engraftment by an increased expression of SDF-1 and osteopontin and, when injected locally, they co-localised around blood vessels in the subendosteal region of mice femurs, restoring BM-niche function even when the stroma is damaged [14].

The activation of specific signalling pathways, such as those dependent on Notch, Wnt/β-catenin molecules [15] or on the adhesion molecules, (fibronectin-1, cadherin-11, vascular cell adhesion molecule-1 (VCAM-1), CX43) and integrins [16] have been recognized to be involved in the interaction between human HSCs and MSCs. The interaction of VCAM-1 with integrin alpha4beta1 through which MSCs modulate the fate of HSCs [17]. Less known is the role of alternative genes and signalling pathways that contribute to regulate stemness, cell proliferation and cell differentiation.

These data strongly suggest that haematological recovery post-ablative chemotherapy or after HSC transplantation depends on and could be improved by the co-operation between MSCs and HSCs. In mice model, Méndez-Ferrer et al. observed that physical association between nestin+MSCs and HSCs is essential to inducing the expression of HSC maintenance genes and to balance HSC quiescence, proliferation and differentiation [13].

In humans, perhaps the best transplant model suggesting the potential role of MSCs in favouring HSC proliferation and differentiation is allogeneic transplantation based on intra-bone injection of cord blood haematopoietic stem cells (CB-CD34+ cells) (IB-HSCT) [18,19]. In this case, positive effects in the haematopoietic recovery observed in the transplanted patients have been attributed to a local and direct interaction of the injected HSCs with other cell components into the BM-niche, including, in particular, with MSCs.

The expectation that an intra-bone co-infusion of MSCs could promote the ability of BM-niche in supporting the post-transplant haematopoiesis by contributing to the repair or replacement of the stromal damage is intriguing but it is not explored. Furthermore, assuming a possible *in vivo* use of MSC and HSC co-infusion, biological aspects regarding the effects of the HSCs on MSCs have not been investigated at all. Since experimental data and comprehensive indications suggesting that HSCs priming with MSCs could improve the clinical outcome in the setting of allogeneic HSCs transplantation are still lacking, we investigated how bone marrow-derived MSCs can affect the molecular and functional phenotype of CB-CD34+ cells and vice versa. To this end, we set up an ex-vivo human MSC-HSC co-culture system in order to evaluate the early proliferative and differentiative effects induced by MSCs on HSCs, and the reciprocal influences on their gene expression profiles.

## Materials and methods

### Ethics statement

Ethics approval to conduct this study was granted by Comitato Etico Provinciale della Provincia di Brescia—ASST Spedali Civili di Brescia (Approval number: NP1871).

### Experimental design

**Fig 1:**
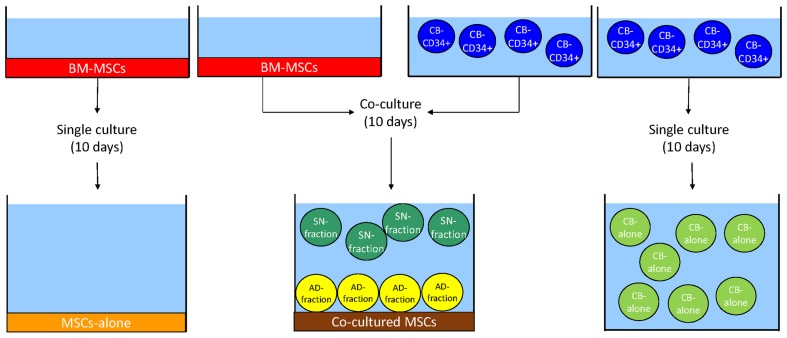
Experimental design of the study. See Material and method section for a complete description of the figure. Ten days of culture were chosen for 2 reasons: first of all, the majority of research articles dealing with MSCs and HSCs range from 7 to 14 days of co-culturing. Secondly, 10 days of co-culturing allow to see a biological effect of MSCs on HSCs without inducing cellular senescence in stromal cells. Indeed, co-cultures were maintained in serum-free medium supplemented with Stem Cell Factor (SCF), Thrombopoietin (TPO) and Granulocyte Colony Stimulating Factor (G-CSF) (see below for details). When MSCs are cultured in this medium for more than 14 days they undergo changes in cell morphology and they begin losing their phenotypic characteristics (data not shown).

BM-derived MSCs (BM-MSCs, red) were seeded on a plastic support. Upon reaching 80% of confluence, previously isolated CD34+ CB-derived HSCs (CB-CD34+ cells, blue) were plated onto a MSCs layer. After 10 days of culture, cells grown in suspension were collected in plastic tubes, while adherent cells were harvested by trypsinization. Two different fractions of HSCs were obtained: the SN-fraction (green), representing the cells grown in the supernatant of the co-cultures, and the AD-fraction (yellow), representing the cells grown directly in contact with the stromal layer, represented by co-cultered MSCs (brown). Single cultures of BM-MSCs and CB-CD34+ cells served as controls, namely MSCs-alone (orange) and CB-alone (light green) (Fig 1).

### CB-CD34+ cell purification

Fresh cord blood (CB) samples were collected after normal vaginal delivery in women without infectious diseases or maternal complications, after informed written consent. Mononuclear cells (MNC) were isolated from 30 ml of CB after density gradient centrifugation using lymphocytes separation media (Histopaque^®^, Sigma-Aldrich) stratification (2000 rpm, 20 min). After washing with Phosphate-Buffered Saline (PBS) (Gibco), MNC were incubated with immunomagnetic beads (CD34+) (Miltenyi) following manufacturer’s instruction. After CD34+ cell recovery, cell purity was assessed by flow cytometry (FACS Canto II^®^, BD) using a panel of CD34-PE and CD45-FITC (BD) fluorochrome-conjugated monoclonal antibodies. TruCount^®^ Absolute-Count Tubes (BD) were used for determining the number of CD34+ cells. The strategy of gating is reported in the S1 and S2 Figs. CB samples were processed within 8 hours from collection; cells with at least 90% of purity were used for co-culture experiments.

### Isolation and characterization of BM-MSCs

Third party-MSCs were obtained from bag washouts after scheduled BM harvests according to standards of National Marrow Donor Program and after informed written consent. The Donor of BM-MSCs was a healthy 20 years old male. Total nucleated cells from filter and bag residues were obtained after two washings with 100–150mL PBS and centrifugation at 200 g for 10 min. The pellet material was gently resuspended in 20–30mL PBS and separated over Histopaque^®^ gradients centrifugation to yield MNC. One million MNC were seeded in a T75 flask (Sarstedt) and cultured in complete ISCOVE’s modified Dulbecco’s medium (Gibco) supplemented with 10% Fetal Bovine Serum (FBS) (Gibco). Medium was changed weekly, discarding non-adherent cells. After 3 weeks, adherent cells were trypsinized and purity was assessed by flow cytometry using a panel of the following antibodies: CD45-FITC, CD90-APC, CD73-PE, CD105-PerCP-Cy5.5 and 7AAD-PerCP (BD). MSCs were also characterized for their differential potential. According to the manufacture’s instruction, cells were cultured in proper media for adipogenic (Mesencult^™^ Adipogenic Differentiation Medium, StemCell Technologies), chondrogenic (StemXVivo^™^ Chondrogenic Differentiation Media, R&D Systems) and osteogenic (Mesencult^™^ Osteogenic Stimulatory Kit, StemCell Technologies) differentation, followed by staining with Oil-Red-O (Sigma Aldrich), Safranin O (Sigma Aldrich) and Alizarin Red S (Sigma Aldrich), respectively. Briefly, 5x10^4^ cells were plated in culture dishes with weekly changing of the specific differentiating media. At the end of the differentiation period, all cultures were fixed with 4% formaldehyde for 30 min and adipocytes were stained with 0,3% Oil Red O solution for 10min; bone cells with 2% Alizarin Red S solution (pH 4.2) for 3 min, while chondrogenic pellets were stained with 0.1% Safranin O solution for 5 minutes (S3 Fig). Once characterized, MSCs (CD45-, CD90+, CD73+, CD105+ cells > 95%) were expanded and stored in liquid nitrogen at the final concentration of 0.5x10^6^ cells/vial. In this study, MSCs were used until third passage.

### CB-CD34+ cells and BM-MSCs co-cultures

BM-MSCs were thawed at 37°C and 1.5x10^6^ cells were plated in three T75 flasks at equal concentrations (0.5x10^6^ cells/flask). Cells were cultured in complete ISCOVE’s modified Dulbecco’s medium supplemented with 10% FBS for a week until 80% confluence.

CB-CD34+ cells were seeded directly onto the MSCs layer at a final concentration of 0.3x10^6^ cells/flask. Cells were maintained in serum-free medium (StemSpan H3000, StemCell Technologies) supplemented with 100 ng/ml Stem Cell Factor (SCF) (Peprotech), Thrombopoietin (TPO) (Peprotech) and Granulocyte Colony Stimulating Factor (G-CSF) (Italfarmaco) for 10 days at 37°C and 5% CO_2_.

A single co-culture experiment consisted of:

- 2 T75 of 0.3x10^6^ CB-CD34+ cells seeded onto MSCs at 80% confluence (CB-CD34+ cells and MSCs co-culture)
- 1 T75 of 0.3x10^6^ CB-CD34+ cells (CB-CD34+ alone)
- 1 T75 of MSCs at 80% of confluence (MSCs alone)

As regards microarray experiments and clonogenic assay, three independent experiments were performed, whereas for the evaluation of CB-CD34+ cell expansion, 5 independent replicates were performed. In all the experiments, cord blood samples were never pooled

### Purification and cell-sorting of BM-MSCs and CB-CD34+ cells

After 10 days of culture, cells grown in suspension were collected in plastic tubes, while adherent cells were harvested by trypsinization. Four different cell fractions were obtained: the single culture of CB-CD34+ cells grown in suspension (CB-alone), the single culture of MSCs grown adherent to the flask (MSCs-alone), the CB-CD34+ cells in the co-culture supernatant (SN-fraction) and the adherent fraction (AD-fraction) of the co-cultures, i.e. adherent MSCs and CB-CD34+ cells grown directly in contact of the stromal layer. Samples were centrifuged 10’ at 1300 rpm, pellets were resuspended in PBS and stained for 30’ on ice with the following fluorochrome-conjugated monoclonal antibodies: CD45-FITC (BD Biosciences), CD73-PE (BD Biosciences), CD90-PE (BD Biosciences), CD105-PE (ImmunoStep), CD34-APC (BD Biosciences), in presence of DAPI (Sigma-Aldrich). Cells were then washed, passed through a 45 um cell strainer (BD Biosciences) and resuspended in PBS. Stained cells were analyzed and sorted by a FACSAria II flow cytometer (Becton Dickinson). Flow cytometric data were analyzed using FACSDiva software (Becton Dickinson). Dead cells and debris were excluded by forward scatter (FSC), side scatter (SSC), and DAPI viability dye. MSCs were identified on the basis of CD73, CD90, CD105 co-expression and lack of CD45. CB-CD34+ cells were identified on the basis of CD45 and CD34 co-expression. At least 0.3x10^6^ gated events were acquired for each sample. Purity of FACS-isolated cells was greater than 95%, as assessed by flow cytometric analysis of post-sorting samples.

### Proliferation and clonogenic assays of CB-CD34+ cells

The proliferation of CB-CD34+ cells was reported as a fold-increase ratio by comparing the number of CB in the Total SN+AD (CD34+ cells grown in presence of MSCs) with the number of CD34+ cells of CB-alone, thus obtaining a readout of the effect of MSCs on the proliferative capacity of the CB-CD34+ cells. Similarly, we reported the fold-increase ratio of CB-CD34+ cells grown in the SN-fraction and in the AD-fraction independently analyzed. We performed 5 independent experiments to achieve statistical significance. The number of human Colony Forming Units (CFUs) of CB-CD34+ cells was analyzed by seeding 1x10^3^ CD34+ cells of CB-, SN- and AD- fractions, respectively, in a 35 mm dish (Sarstedt). Seeded cells were cultured in MethoCult^™^ H4535 methylcellulose media (StemCell Technologies) at 37°C, in humidified atmosphere and 5% CO_2_. After 14 days, the number of erythroid burst-forming unit (BFU-E), granulocyte-macrophage colony forming unit (CFU-GM) and granulocyte/erythrocyte/macrophage/megakaryocyte colony forming unit (CFU-GEMM) was determined with manual count at optical microscope. Values came from three replicates.

### RNA extraction and microarray analysis

Total cellular RNA was isolated using Qiagen total RNA purification kits as recommended by the manufacturer (Qiagen). RNA integrity, purity and concentration were then verified by the Bio-Analyzer technique (Applied Biosystem) and NanoDrop 2000 spectrophotometer (Life Technologies). Three independent experiments for each culture condition were performed. An amount of 50 ng of RNA was converted in labelled aRNA using the GeneChip 3’ IVT Expression kit (Affymetrix) and then used to hybridized the Affymetrix HG-U133A PLUS 2.0 GeneChip arrays that were subsequently washed and stained using the GeneChip Hybridization, Wash and Stain Kit (Affymetrix). Genechips were then analyzed with the Affymetrix Scanner 3000 and the images obtained were processed by Microarray Analysis Suite 5.0 (MAS 5.0) algorithm. Hybridization quality, absolute call (absent or present) and mRNA quantification were also evaluated.

The complete dataset has been submitted to the gene expression omnibus data (GEO) public database at NCBI, and the accession number is GSE90970.

### Gene validation by quantitative RT-PCR

Quantitative RT-PCR (qRT-PCR) was carried out by a Light Cycler 480 sequence detection system (Roche Diagnostics) on total RNAs (100 ng) reversely transcribed using the High Capacity cDNA Archive Kit (Applied Biosystems), according to the manufacturer’s instructions. Each cDNA sample was run in duplicate for either each target (*VEGFA* and *CCNA1*) or *GAPDH* endogenous control. TaqMan gene assays, supplied as ready solutions, and TaqMan Universal PCR Master Mix were provided by Applied Biosystems. Quantification of qRT-PCR signals was performed using the (2^-Delta DeltaCt^) method [20], which calculates relative changes in gene expression of the target gene normalized to the *GAPDH* endogenous control and relative to a calibrator sample. The values obtained were represented as relative quantity of mRNA level variations.

### Bioinformatic analysis

Number of CB-CD34+ cells and clonogenicity assays were presented as mean ± standard deviations (SDs) and analyzed using Student's t-test. Differences between the samples were considered statistically significant for p <0.05.

Microarrays data were processed by using Partek^®^ Genomics Suite^®^ software to identify genes differentially expressed in MSCs and CB-CD34+ cells following co-culture. The lists of genes were obtained by applying multiple comparison tests (Bonferroni’s correction and False Discovery Rate) after ANOVA analysis, and only genes with a fold change <-2 or> +2 were selected.

The analysis of molecular pathways was carried out as previously reported [21–24]. Briefly, EASE software was applied to establish whether specific cell functions and biological processes, defined according to gene ontology, were significantly represented among the deregulated genes through the DAVID Functional Annotation Bioinformatics Microarray Analysis [25,26]. Broad Institute Gene Set Enrichment Analysis (GSEA) software was performed to identify significant enrichments in differentially expressed genes signatures, in case of nominal P values corrected for False Discovery Rate (FDR) for enrichment scores minor than 0.05. Specifically, GeneOntology Biological Processes, Oncogenic pathways, immune pathways, and curated gene sets were considered [27]. The network analyses were generated through the use of Ingenuity Pathway Analysis^®^ (IPA^®^, Qiagen).

## Results

### Co-cultures of CB-CD34+ cells and MSCs

**Fig 2:**
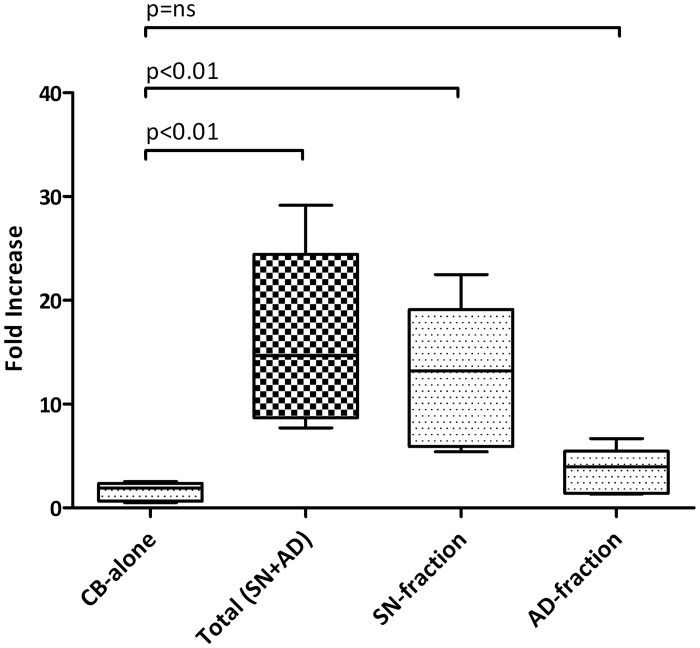
*Ex vivo* expansion of CB-CD34+ cells with MSCs. 5×10^5^ CB-CD34+ cells were cultured alone or in presence of a layer of MSCs for 10 days; the fold increase in total cell number was calculated from the original CD34+ cells seeded at day 0. The absolute number of CB-CD34+ cells was measured by flow cytometry. Values came from 5 independent replicates. Abbreviation: CB-alone: number of CB-CD34+ cells after 10 days of single culture; SN-fraction: CB-CD34+ cells in the supernatant (SN-fraction) of the co-cultures with MSCs; AD-fraction: CB-CD34+ cells grown directly in contact with MSCs layer; Total SN+AD: total number of CB-CD34+ cells after co-culture with MSCs.

Co-culturing experiments were performed to evaluate the MSCs’ effects on the proliferation and differentiation of CB-CD34+ cells. After 10 days, when cultured alone (CB-alone), 0.5x10^6^ CB-CD34+ cells were obtained. The ratio of CD34+ cells grown in single culture (CB-alone) was compared to the total number of CD34+ cells after co-culture with MSCs (Total SN+AD). A fold-increase of 14.68 (median of 5 replicates, p = 0.019) was found. Notably, a 13.20-fold increase in the number of CD34+ cells was observed with the sorted SN-fraction (median of 5 replicates, p = 0.025), while no statistically significant difference was found with the sorted AD-fraction. Data are summarised in Fig 2 and in S1 Table.

**Fig 3:**
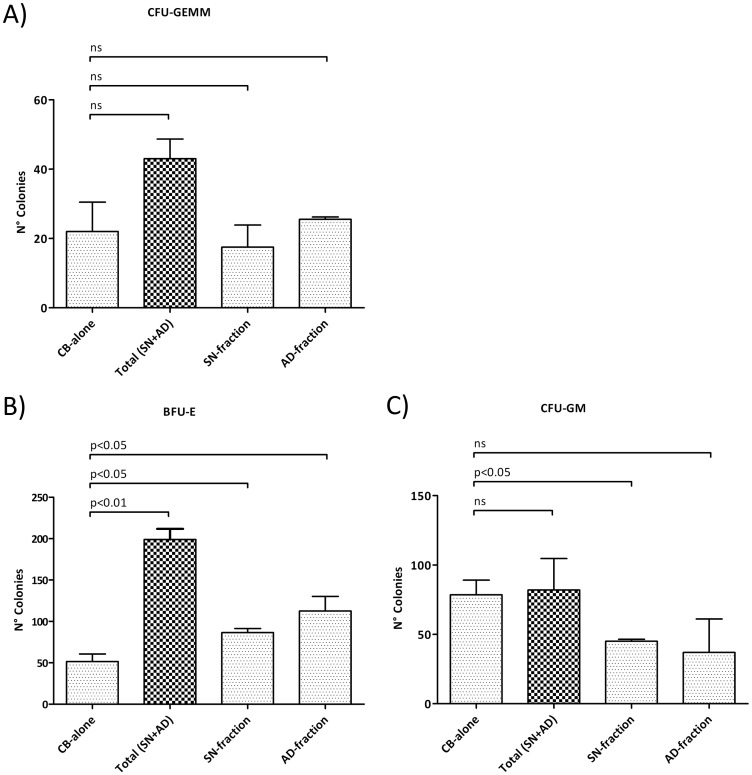
Clonogenic assays of CB-CD34+ cells. Results of statistical analysis of methylcellulose-based clonogenic assay performed on CB-CD34+ cells plated after 14 days of co-culture with human MSCs. (A) CFU-GEMM count; (B) BFU-E count; (C) CFU-GM count. Values are reported as mean ± SD. The results derive from three independent experiments. Abbreviations: BFU-E, Burst forming unit-erythroid; CFU, Colony forming unit; E, erythrocyte; GM, granulocyte-monocyte; GEMM, granulocyte-erythrocyte-monocyte-megakaryocyte.

The colony-forming unit ability of CB-CD34+ cells was investigated by performing a clonogenic assay in methylcellulose-based cultures. A statistically significant difference in clonogenic capacity was found by comparing CB-alone-derived colonies with the co-culture-derived colonies (Total SN+AD, SN-fraction or AD fraction alone). In particular, the methylcellulose-based assay showed no statistically significant differences in CFU-GEMM number, while a significant increase in the BFU-E colonies was observed in co-culture-derived fractions (Total SN+AD p<0.01, SN-fraction p<0.05 or AD-fraction p<0.05), and this was combined with a decrease in the CFU-GM, in the SN-fraction only (p<0.05). Data are shown in Fig 3.

### Gene expression profiling of CB-CD34+ cells after co-culture with MSCs

CB-CD34+ cells, either cultured alone or with MSCs (Total SN+AD, SN-fraction or AD-fraction alone), were analysed for their gene expression profile by using GeneChip arrays HG-U133A 2.0 PLUS (Affymetrix). First, gene expression profiles of SN-fraction vs. AD-fraction were analysed to assess possible differences in their transcriptomes. Since no statistically significant differences were found between SN- and AD- fractions (data not shown), CB-alone and Total SN+AD (SN- and AD- fractions together) were compared.

**Fig 4:**
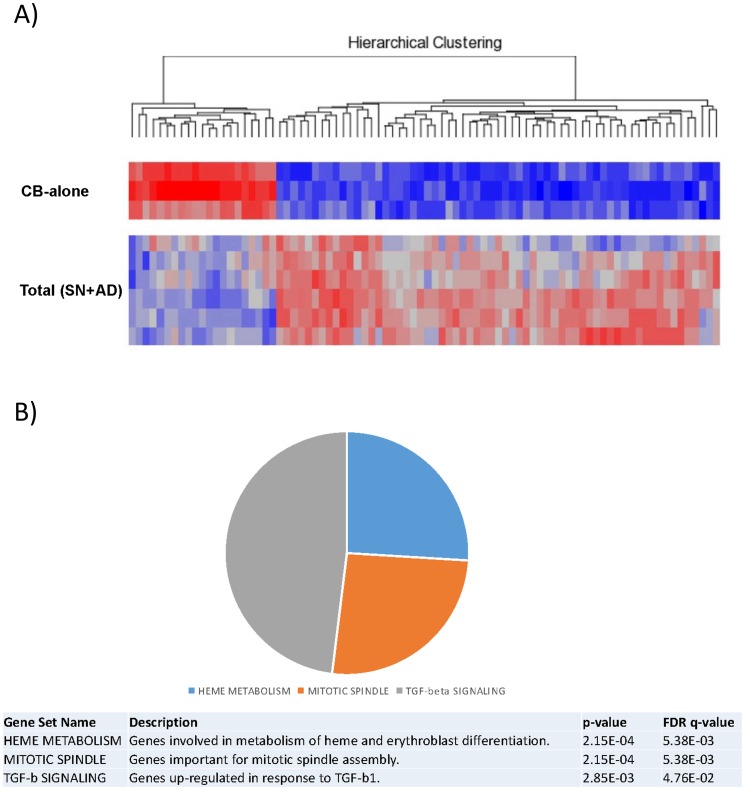
Gene expression analysis of CB-CD34+ cells after co-culture with MSCs. (A) Supervised analysis of CB-alone vs. Total SN+AD. CB-CD34+ cells were maintained for 10 days in co-culture with MSCs. A heat map of the genes differentially expressed in a statistically significant manner after co-culture (corrected p value <0.05) is plotted. (B) Gene Set Enrichment Analysis (GSEA) of CB-alone vs Total SN+AD. Enriched gene sets from hallmark gene sets are plotted. Please refer to S2 Table for the complete GSEA details. Abbreviation: CB-alone: CB-CD34+ cells in single culture; Total SN+AD: CB-CD34+ cells grown in presence of MSCs.

Seventy-one out of 47,000 genes spotted on the array were expressed differentially, with a corrected p<0.05 coupled with a fold change < -2 or > +2. Based on the expression of such 71 genes, CB-alone and culture-derived cells (AD+SN) appeared clearly distinct (Fig 4A).

**Fig 5:**
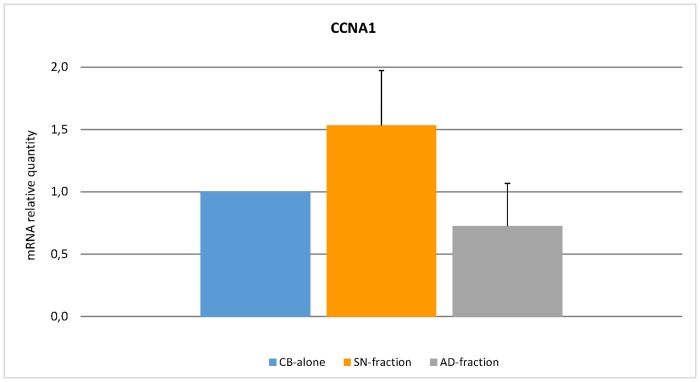
Real-Time Quantitative Polymerase Chain Reaction PCR (qRT-PCR) analysis of *CCNA1* gene. Expression level of *CCNA1* gene was assessed by qRT-PCR. Quantification of mRNA relative quantity was calculated as relative changes in gene expression of the target gene normalised to the *GAPDH* endogenous control and relative to a calibrator sample. The values obtained were represented as relative quantity of mRNA level variations.

To understand better the possible biological role of the transcriptional differences induced by culture, we investigated whether the identified gene signature was enriched with genes involved in specific cellular programs and functions. Through gene set enrichment analysis (GSEA), we found a significant enrichment in genes involved in heme metabolism, mitotic spindle formation and proliferation, and TGF-beta signalling (Fig 4B, S2 Table). In addition, IPA-based pathway analysis revealed the significant involvement of genes regulating cell-to-cell signalling, haematological system development and function, cell cycle, growth and proliferation (S3 Table). In particular, certain genes (*e*.*g*. *ID1* and *PCGF2*) appeared to be key regulators of several processes affecting haematopoiesis (S4–S6 Figs). To confirm the microarray data, qRT-PCR was carried out on *CCNA1* that resulted differentially expressed between CB-alone versus SN-fraction and AD-fraction. Data related to the relative quantity of mRNA level variations are recorded in Fig 5.

### Gene expression profiles analysis of MSCs after co-culturing with CB-CD34+ cells

Co-culture effects on MSC transcriptome were then studied. To this aim, microarray analysis was carried out on either MSCs-alone or MSCs maintained for 10 days in co-culture with CB-CD34+ cells. In the latter case, only MSC samples with at least 95% purity following cell sorting were used.

**Fig 6:**
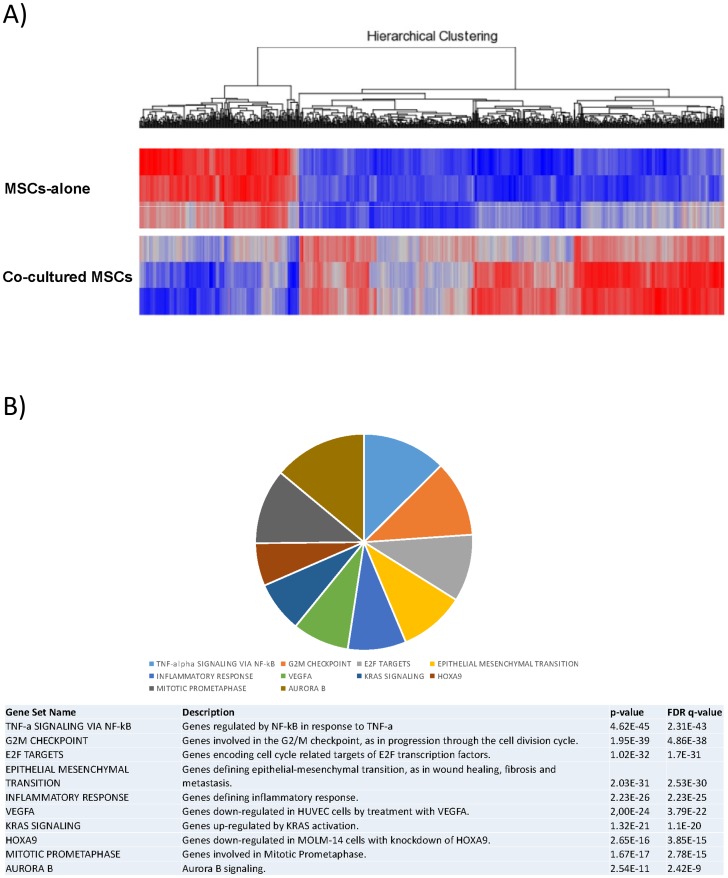
Gene expression profiles analysis of MSCs after co-culturing with CB-CD34+ cells. (A) Supervised analysis of MSCs-alone vs. MSCs after 10 days of co-culture with CB-CD34+ cells. A heat map of the genes differentially expressed in a statistically significant manner after co-culture (corrected p value <0.05) is plotted. (B) Gene Set Enrichment Analysis (GSEA) of MSCs-alone vs. MSCs maintained for 10 days in co-culture with CB-CD34+ cells. Top 10 gene sets from hallmark gene sets, canonical/KEGG pathways and oncogenic signatures are plotted. Please refer to S4 Table for the complete GSEA details.

Five-hundred-ninety-seven genes were expressed differentially in untreated vs. cultured MSCs with a corrected p<0.05 and a fold change < -2 or > +2. The two cell populations were clearly separated as suggested by hierarchical clustering based on the expression of these genes (Fig 6A).

Again, to explore the potential biological role associated with the transcriptional changes, GSEA and pathway analyses were performed. Among others, we found a significant enrichment in genes involved in angiogenesis and VEGF signalling, proliferation and aurora kinase B activity, and NF-kappa B pathway (Fig 6B, S4 Table), as well as genes involved in cell-to-cell signalling and interaction, cell cycle, and cardiovascular system development and function (S7 and S8 Figs; S5 Table).

**Fig 7:**
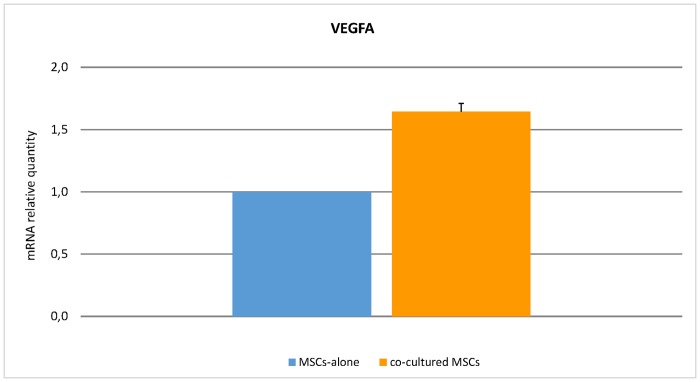
Real-Time Quantitative Polymerase Chain Reaction PCR (qRT-PCR) analysis of *VEGFA* gene. Expression level of *VEGFA* in co-cultured MSCs was assessed by qRT-PCR. Quantification of mRNA relative quantity was calculated as relative changes in gene expression of the target gene normalised to the *GAPDH* endogenous control and relative to a calibrator sample. The values obtained were represented as relative quantity of mRNA level variations as compared to MSCs-alone.

Among the genes differentially expressed in MSCs-alone vs. co-cultured MSCs, *VEGFA* was used as validating gene of microarray data. QRT-PCR analysis confirmed a significant *VEGFA* up-regulation following co-culture of MSCs and HSCs (Fig 7).

## Discussion

MSCs are essential for HSC self-renewal and proliferation [28–30] and, in the case of IB-HSCT, it has been suggested that they can favour haematologic recovery by interacting with CB-CD34+ cells when injected locally into the BM [31,32]. Nevertheless, experimental data and comprehensive indications suggesting that HSCs priming with MSCs could improve the clinical outcome in the setting of allogeneic HSCs transplantation are still lacking. For this purpose, we investigated how bone marrow-derived MSCs can affect the molecular and functional phenotype of CB-CD34+ cells and vice versa. To this end, we set up a co-culture system of human CB-CD34+ cells with third-party MSCs to elucidate the mechanisms depending on the early reciprocal interaction between MSCs and HSCs.

In presence of BM-MSCs, a significant increase in CB-CD34+ cell proliferation (fold increase of 14.68, p = 0.019) was observed as compared to the CB-CD34+ cells cultured alone. Since the increase was mainly observed in the SN-fraction (fold increase of 13.20, p = 0.025), we supposed that the inductive proliferative effects of MSCs on HSCs could be mediated by MSCs-derived molecules. We did not carry out a systematic and complete study of secretome. It has been already reported that several cytokines and growth factors, such as SDF-1, stem cell factor (SCF), Flt-3 ligand (FL), thrombopoietin (TPO), interleukin (IL)-6, IL-11, tumor necrosis factor-alpha (TNF-alpha), and transforming growth factor-beta 1 (TGF-beta1), are involved in inducing proliferative effects on HSCs [33]. We looked at the levels of TNF-alpha, interferon-gamma, IL-8, IL-6, sVCAM-1 and sICAM-1 in the supernatant of the cultures, and at the end of 10 days of co-culture, we did not find a significant increase in the levels of the abovementioned cytokines in the co-culture medium, even though higher detectable levels of TNF-alpha, IL-6 and sVCAM-1 were present (data not shown). Although a possible partial degradation of these cytokines cannot be excluded, our results suggest that their production was not induced by co-culturing. This observation is not in contrast with that which has been reported in a number of studies published in recent years [34–40]. The majority of these studies did not specify whether the inductive proliferative effects of MSCs on HSCs depended on direct contact with MSCs or mediation by soluble molecules. Furthermore, they did not provide any information regarding HSC differentiation, as well as gene expression profiles. Alakel et al. investigated the impact of direct contact between MSCs and HSCs in terms of expansion, differentiation, migratory capacity, and gene expression profile [41]. However, they used mobilised CD133+ haematopoietic progenitor cells from healthy adult donors instead of CB-CD34+ cells collected after natural delivery, thereby introducing the granulocyte colony-stimulating factor (G-CSF) treatment as a variable. Nevertheless, their results are superimposable with ours; in particular, they observed that when CD133+ cells were grown in the presence of MSCs, there was a significant increase in the number of haematopoietic progenitor cells and an increase in the number BFU-E colonies. Moreover, when they performed gene expression analysis between non-adherent and adherent fractions, they observed a modulation in genes involved in cell-to-cell and cell-to-extracellular matrix contact, cell cycle and some signalling pathways. Interestingly, just like Alakel et al. in their study, we found that co-cultured CB-CD34+ cells were first induced by MSCs towards erythroid commitment. On the other hand, we did not observe an inductive differentiation effect on CFU-GM. We do not have a clear explanation as to why the BFU-E differentiation was favoured as compared to CFU-GEMM and CFU-GM. In this regard, we can only speculate that the preferential shift towards erythroid commitment is a priority and thus the first and more important step for initiating new haematopoietic reconstitution. In this regard, Alakel et al. also did not provide a clear explanation of this phenomenon.

The increased capacity of proliferation and the preferential erythroid differentiation of CB-CD34+ cells co-cultured with MSCs were confirmed by gene expression profile analysis. IPA-based pathway analysis revealed significant involvement of genes regulating cell-to-cell signalling (e.g. *ARHGEF2*, *DGKE*) [42,43], haematological system development and function (e.g. *TPP2*, *HOXA9*, *SOS1*) [44–46], cell cycle, growth and proliferation (e.g. *AIM2*, *BMP2K*, *ZNRF3*) [47,48]. In particular, genes such as *ID1* and *PCGF2* should be deemed relevant since they are recognised to be the key regulators of several processes affecting haematopoiesis [49–51]. Furthermore, the higher expression of *CCNA1* gene, which encodes for cyclin A1, a master regulator of the cell cycle [52], would confirm the higher proliferation rate of HSCs in the presence of MSCs. The enrichment in genes involved in erythroid differentiation (e.g. *LAMP2*, *CLCN3*, *BMP2K* and *SLC30A1*) [53–56] and down-modulation of genes participating to myeloid and lymphoid differentiation (e.g. *ID1*, *PCGF2*) [57,58] are in accordance with the preferential shift of HSCs towards the erythroid commitment observed in the clonogenic assays. Interestingly, bioinformatic analysis did not reveal a modulation of genes encoding haemoglobin proteins (such as HBA1, HBA2, HBB, HBG1, HBG2). This could be explained by the fact that gene expression profile analyses in co-cultured CB-CD34+ cells were performed after cell sorting on the basis of CD45 and CD34 expression. In this setting, only immature haematopoietic progenitors were examined. Since genes encoding haemoglobin proteins get expressed during erythrocyte differentiation, it is unlikely to record their statistically significant differences in terms of gene expression. Indeed, only HBB gene showed a borderline significant trend (FDR = 0.08, fold change = 1.2).

Not only did the MSCs reveal to be able to modulate both the expansion of CB-CD34+ cells (particularly in the SN-fraction) and the commitment towards erythroid lineage, but it was also found that HSCs influence MSCs behaviour. Unlike Alakel's study, where authors fucused on hematopoietic compartment only, we also performed a deep characterization of the stromal component. Interestingly, MSCs co-cultured with CB-CD34+ cells revealed a modulation of genes involved in molecular pathways related to hypoxia and oxygen-level response (e.g. *TNFAIP3*, *SLC2A3*, *KLF6*) and also a profound modification of pathways involved in angiogenesis and vascular processes (e.g. *VEGFA*, *IGF1*, *ID1*) [59–64], as well as *mTORC1* [65,66] and Aurora kinases A and B [67,68]. Among these genes, *VEGFA* is one of the most intriguing because it is an inducible growth factor that stimulates the formation of new blood vessels in response to hypoxia, and it is one of the mechanisms by which MSCs can be involved in tissue regeneration [69–71]. In particular, VEGFA is reported to stimulate postnatal haematopoiesis [72] and to make stem cells resistant to an ischemic environment they would encounter after transplantation into injured tissue and it improves the survival of both the transplanted cells and the host cells at the injury site [73]. The qRT-PCR analysis on *VEGFA* confirmed its up-regulation, in agreement with the microarray results. These data are thus indicative of a bi-directional interaction between CB-CD34+ cells and MSCs that would favour the remodelling of the BM-niche structure, and underline the importance of a switch from the osteogenic to the vascular compartment in transplantation.

## Conclusion

Taken all together, our results suggest that MSCs can exert a priming effect on CB-CD34+ cells, possibly by secreting specific molecules essential to the regulation of HSC proliferation and erythroid differentiation. In turn, CB-CD34+ cells seem to be able to polarise the BM-niche towards the vascular compartment by modulating molecular pathways related to hypoxia and angiogenesis. From a clinical point of view, since it is known that in the allo-transplanted patients MSCs may decrease by up to 90% and that this reduction is correlated to a defective haematopoietic reconstitution [74], these data would suggest that a co-infusion of MSCs with CB-CD34+ cells may favour the haematological recovery after IB-HSCT, possibly by replacing damaged stroma and by promoting HSC engraftment through the remodelling the BM-niche from the osteogenic compartment to the vascular one.

## Supporting information

S1 Fig Flow cytometry detection of CB-CD34+ cells. HSCs were considered to be CD45+ ^dim^ CD34+.
(TIFF)

S2 Fig Flow cytometry detection of CB-CD34+ cells after immunomagnetic enrichment. (TIFF)

S3 Fig Characterisation of BM-derived MSC differentiation potential. Osteogenic, chondrogenic and adipogenic differentiation was assessed after culture in proper media and specific staining.
(TIFF)

S4 Fig IPA analysis on CB-CD34+ cells co-cultured with MSCs. The network is representative of cell cycle, cell death and survival pathways.
(TIFF)

S5 Fig IPA analysis of CB-CD34+ cells co-cultured with MSCs. The network is representative of cellular growth and proliferation, haematological system development and function and haematopoietic pathways.
(TIFF)

S6 Fig IPA analysis of CB-CD34+ cells co-cultured with MSCs. The network is representative of cell-to-cell signaling and interaction pathways.
(TIFF)

S7 Fig Schematic representation of the cellular growth and proliferation network as identified by IPA pathway analysis and specifically related to hematopoiesis. Key regulator genes are highlighted by blue circles.
(TIFF)

S8 Fig IPA analysis of MSCs after co-culture with CB-CD34+ cells. The plot is representative of cell-to-cell signaling and interaction, cellular movement, and immune cell trafficking networks.
(TIFF)

S9 Fig IPA analysis of MSCs after co-culturing with CB-CD34+ cells. The plot is representative of cardiovascular system development and function, cellular development and haematological system development and function networks.
(TIFF)

S1 Table *Ex vivo* expansion of CB-CD34+ cells with MSCs. The table summarise the results about the assessment of the *ex vivo* expansion of CB-CD34+ cells grown in presence of MSCs. Briefly, 5×10^5^ CB-CD34+ cells were cultured alone or in presence of a layer of MSCs for 10 days; the fold increase in total cell number was calculated from the original CD34+ cells seeded at day 0. Absolute number of CB-CD34+ cells was measured by flow cytometry. Values derive as median of 5 replicates. Abbreviation: CB-alone: number of CB-CD34+ cells after 10 days of single culture; SN-fraction: CB-CD34+ cells in the supernatant (SN-fraction) of the co-cultures with MSCs; AD-fraction: CB-CD34+ cells grown directly in contact with MSCs layer; Total SN+AD: total number of CB-CD34+ cells after co-culture with MSCs.
(DOC)

S2 Table Gene Set Enrichment Analysis (GSEA) of CB-alone vs. total SN+AD. The table reports Hellmark Genes, Oncogenic-linked Genes, Immune-linked Genes and Pathways enriched after co-culture. Each category is reported in a different sheet, where the Gene Set Name, the number of Genes in Gene Set (K), the Description, the number of Genes in Overlap (k), the k/K ratio, the p-value and the FDR q-value are listed. The Gene/Gene set overlap Matrix reports also the Entrez Gene ID, the Gene Symbol and the Description fully detailed.
(XLS)

S3 Table Ingenuity Pathway Analysis (IPA) of CB-alone vs. total SN+AD. The table reports the networks in which the differentially expressed genes (in bold characters) are involved. Columns list the Molecules in Network, the Score, the Focus-molecules and the direct related Top Diseases and Functions Molecules.
(DOCX)

S4 Table Gene Set Enrichment Analysis (GSEA) of MSCs-alone vs. co-cultured MSCs. The table reports Hellmark Genes, Oncogenic-linked Genes, Immune-linked Genes and Pathways enriched after co-culturing. Each category is reported in a different sheet, where the Gene Set Name, the number of Genes in Gene Set (K), the Description, the number of Genes in Overlap (k), the k/K ratio, the p-value and the FDR q-value are listed. The Gene/Gene set overlap Matrix reports also the Entrez Gene ID, the Gene Symbol and the Description fully detailed.
(XLS)

S5 Table Ingenuity Pathway Analysis (IPA) of MSCs-alone vs. co-cultured MSCs. The table reports the networks in which the differential expressed genes (in bold characters) are involved. Columns list the Molecules in Network, the Score, the Focus-molecules and the direct related Top Diseases and Functions Molecules.
(DOCX)
