# Supplementary figures and images for: Mesenchymal stromal cells (MSCs) induce ex vivo proliferation and erythroid commitment of cord blood haematopoietic stem cells (CB-CD34+ cells)
Source: PLoS One. 2017 Feb 23;12(2):e0172430. doi: 10.1371/journal.pone.0172430 (PMC5322933; doi:10.1371/journal.pone.0172430)

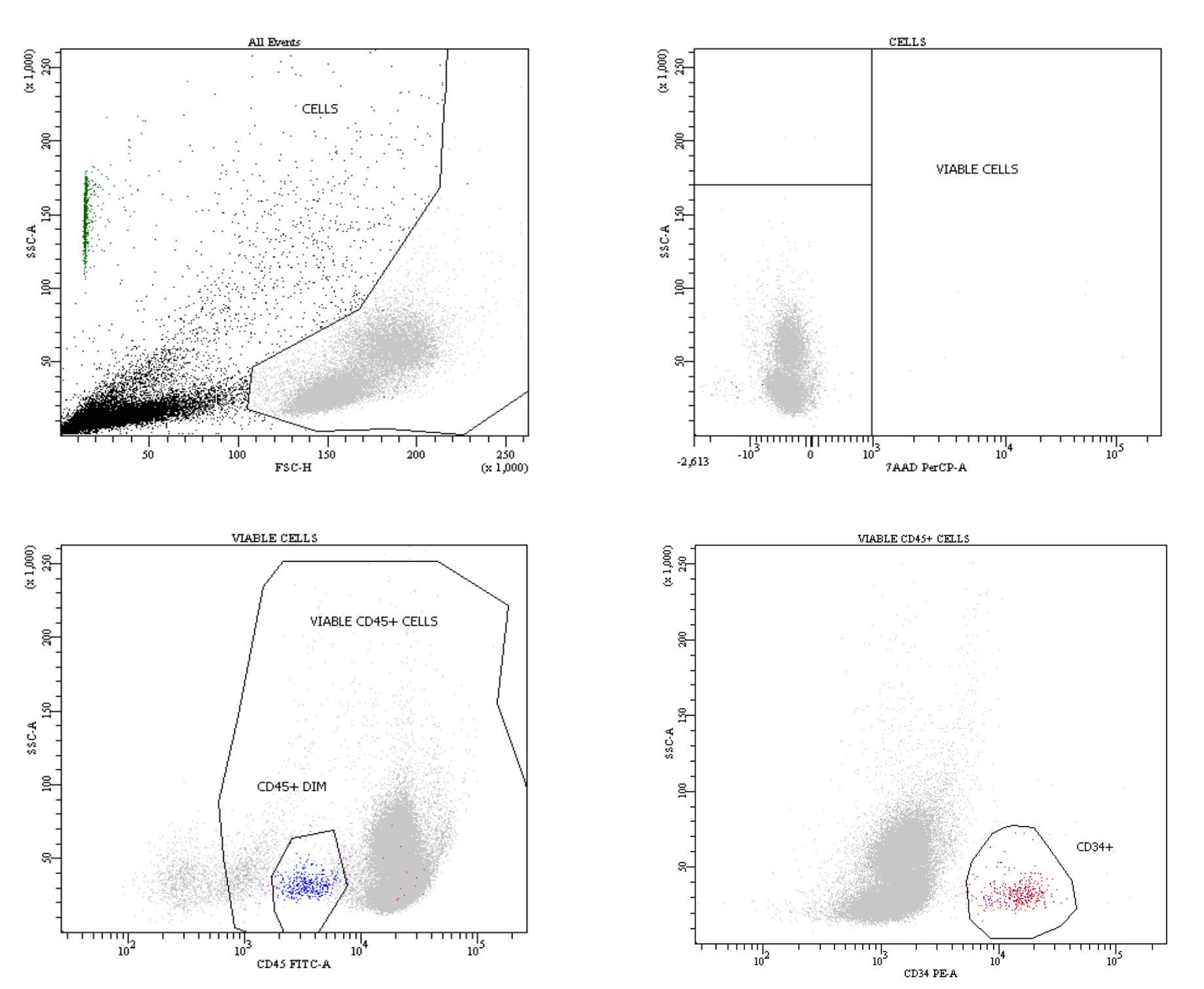

Supplement: S1 Fig — HSCs were considered to be CD45+ dim CD34+. (TIFF) [file pone.0172430.s001.tiff]

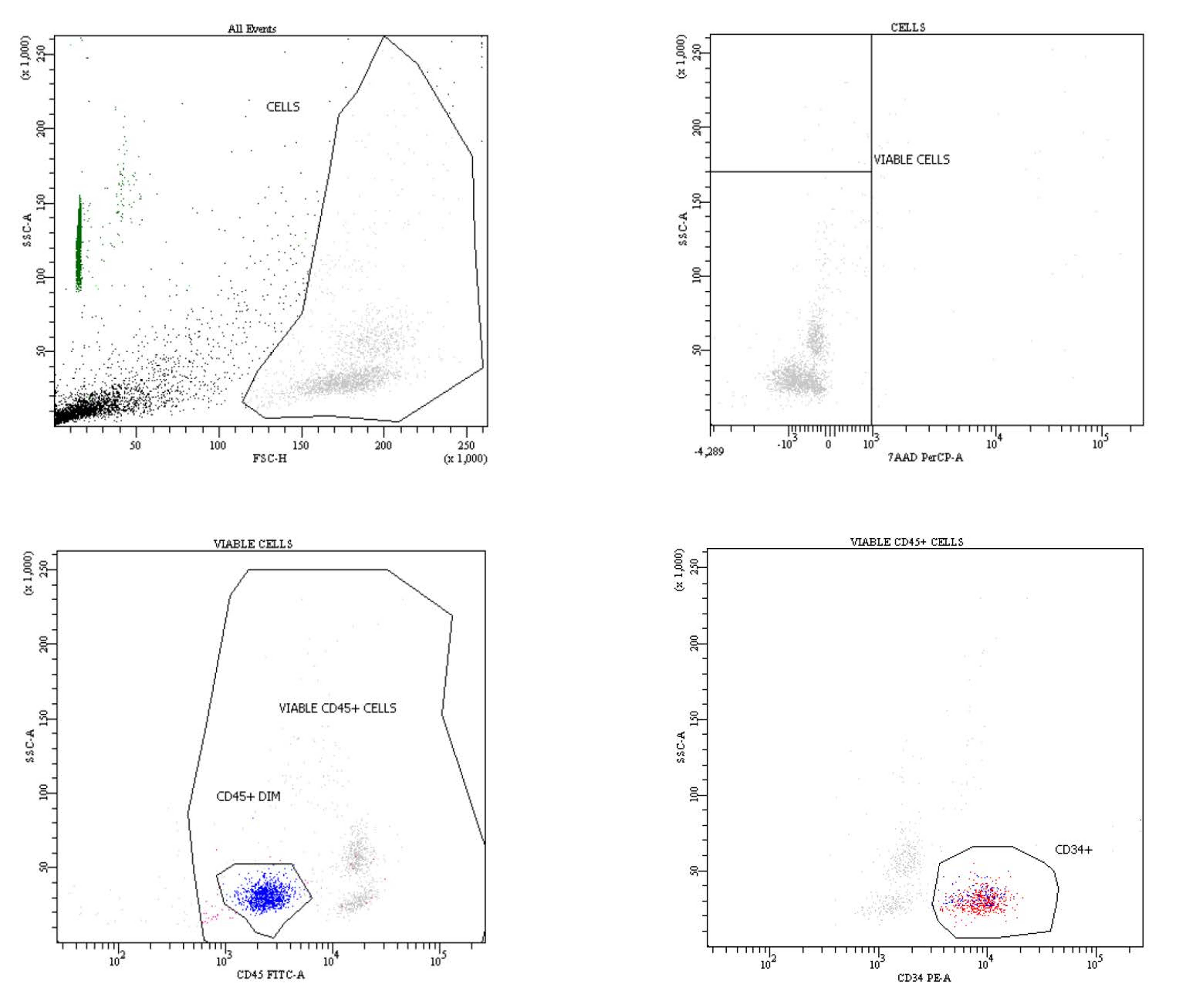

Supplement: S2 Fig — (TIFF) [file pone.0172430.s002.tiff]

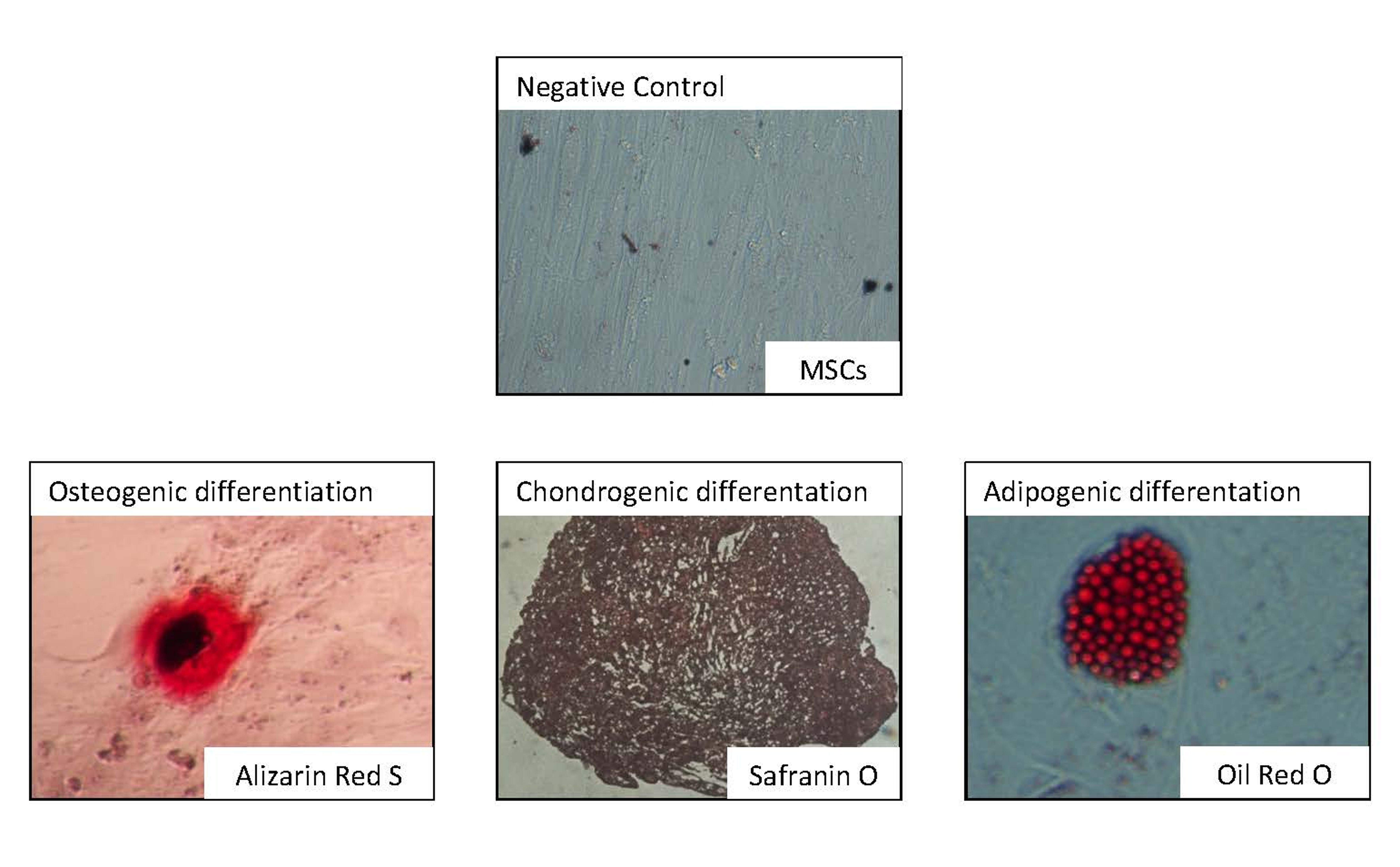

Supplement: S3 Fig — Osteogenic, chondrogenic and adipogenic differentiation was assessed after culture in proper media and specific staining. (TIFF) [file pone.0172430.s003.tiff]

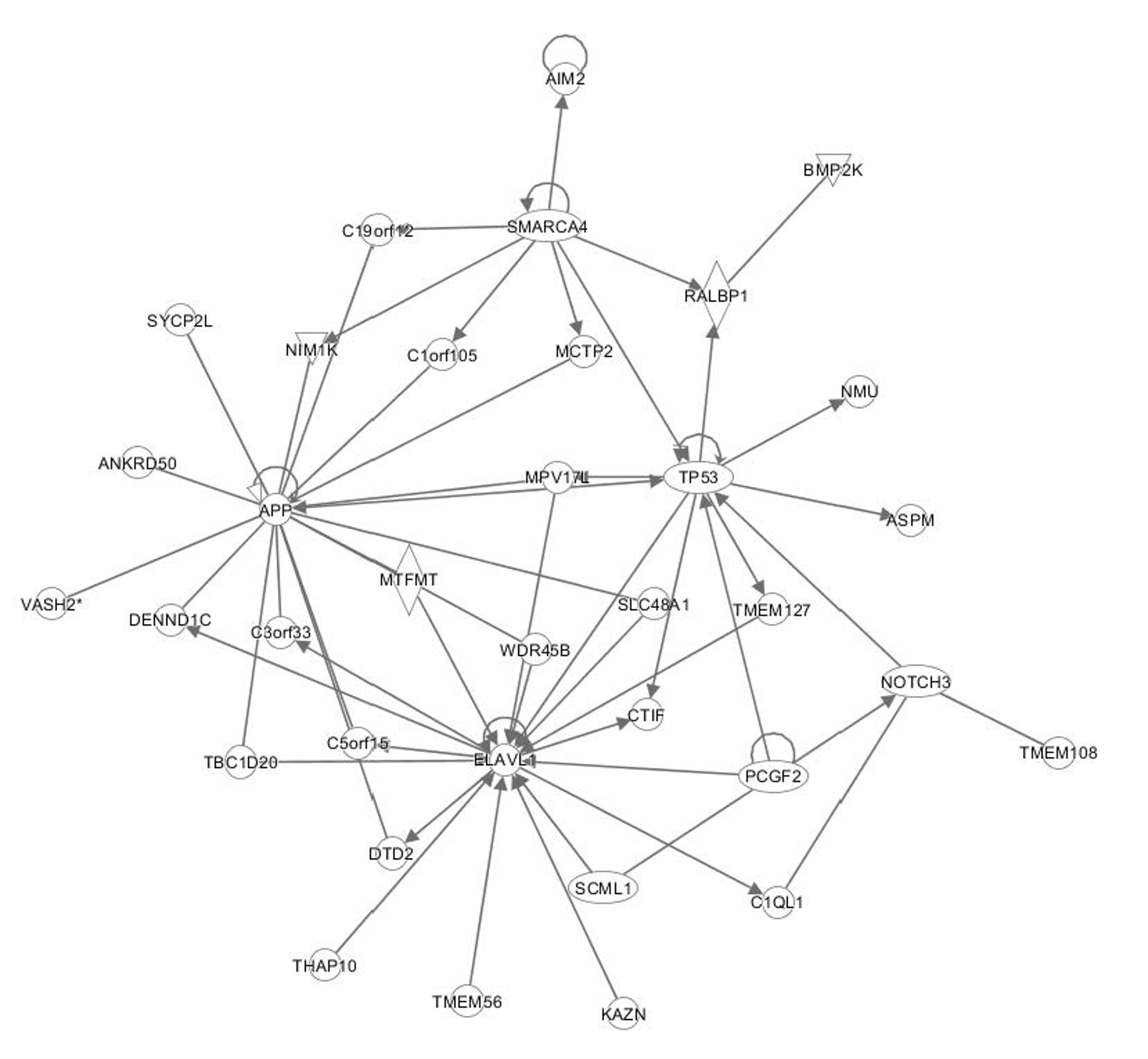

Supplement: S4 Fig — The network is representative of cell cycle, cell death and survival pathways. (TIFF) [file pone.0172430.s004.tiff]

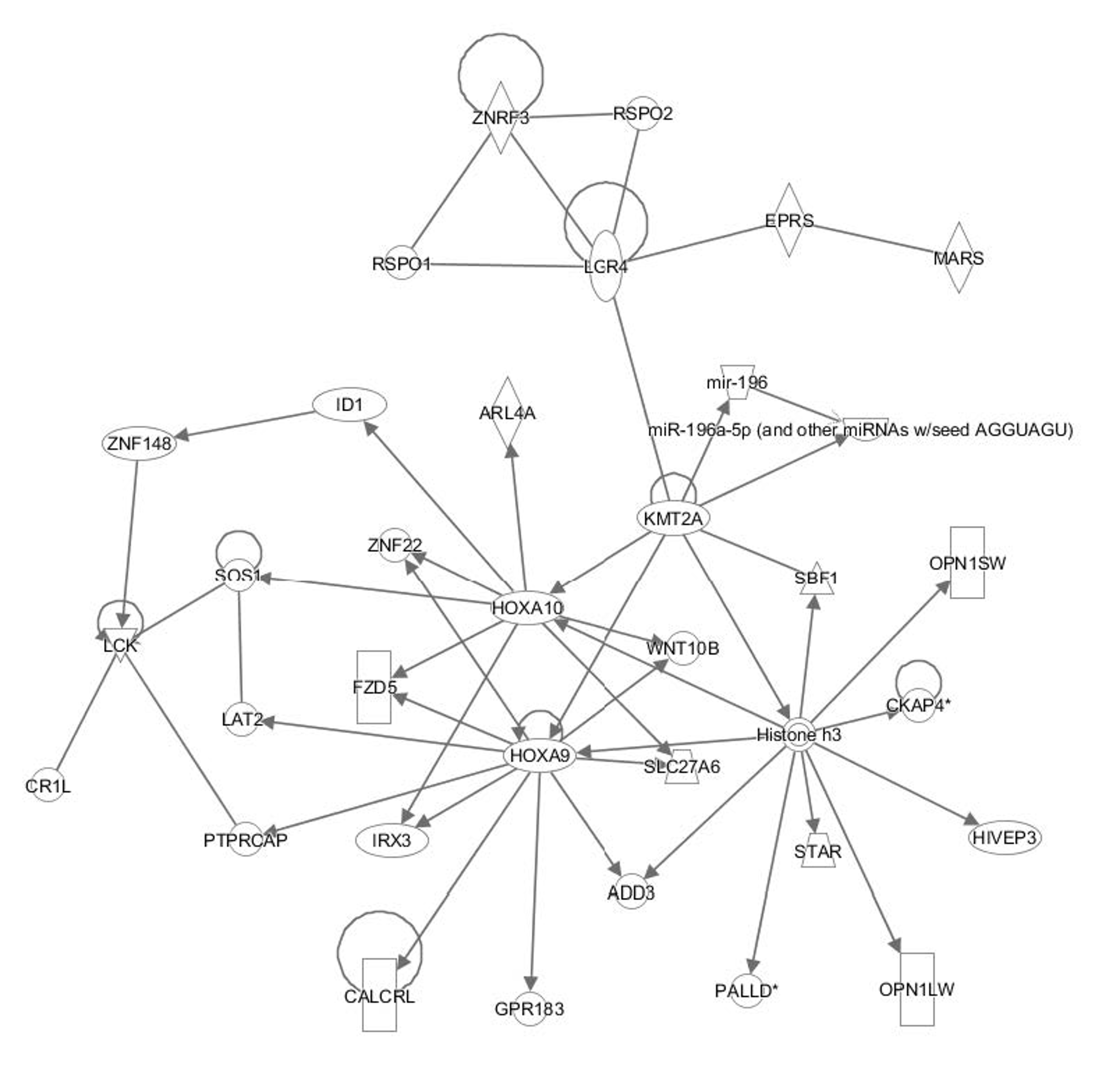

Supplement: S5 Fig — The network is representative of cellular growth and proliferation, haematological system development and function and haematopoietic pathways. (TIFF) [file pone.0172430.s005.tiff]

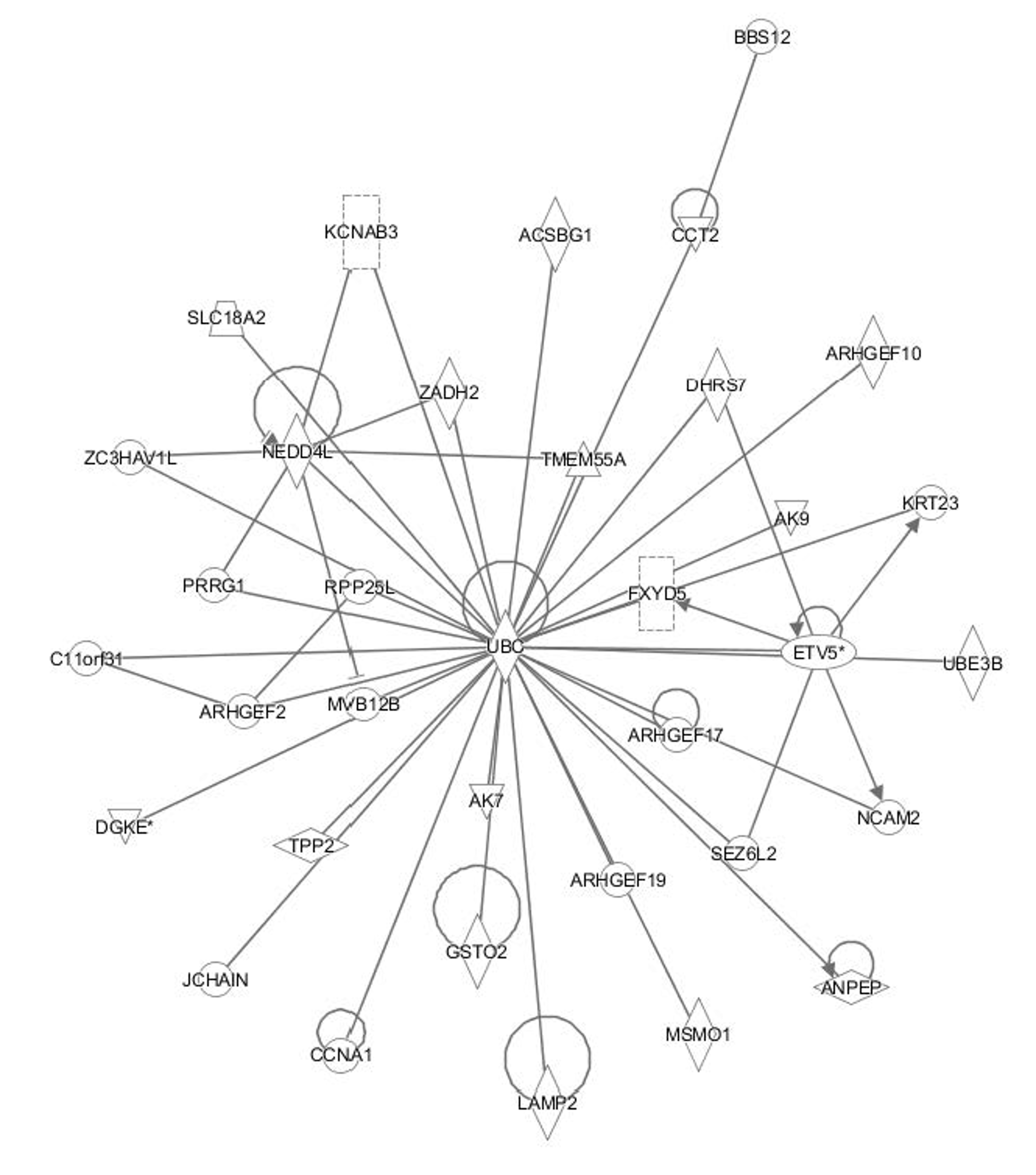

Supplement: S6 Fig — The network is representative of cell-to-cell signaling and interaction pathways. (TIFF) [file pone.0172430.s006.tiff]

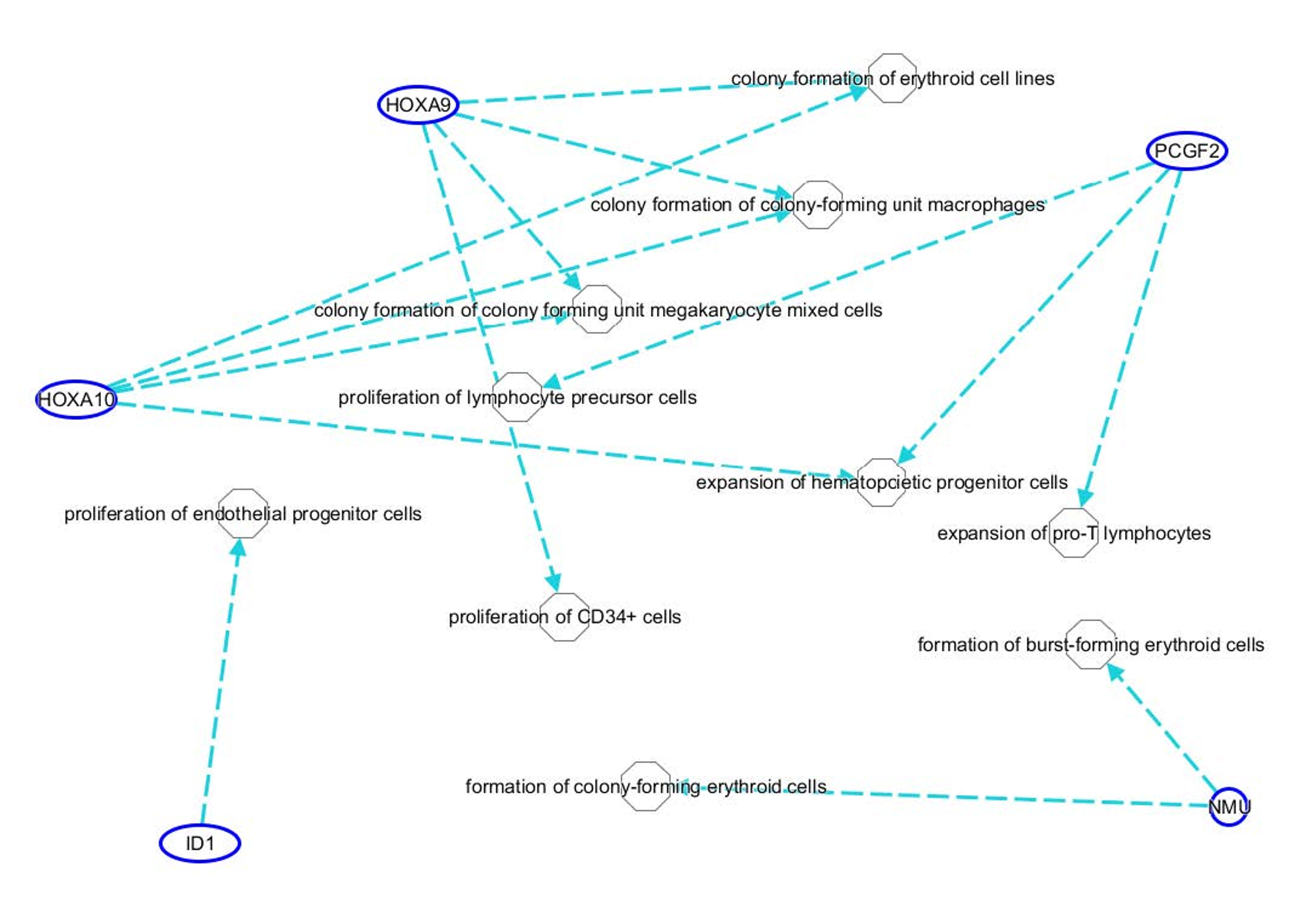

Supplement: S7 Fig — Key regulator genes are highlighted by blue circles. (TIFF) [file pone.0172430.s007.tiff]

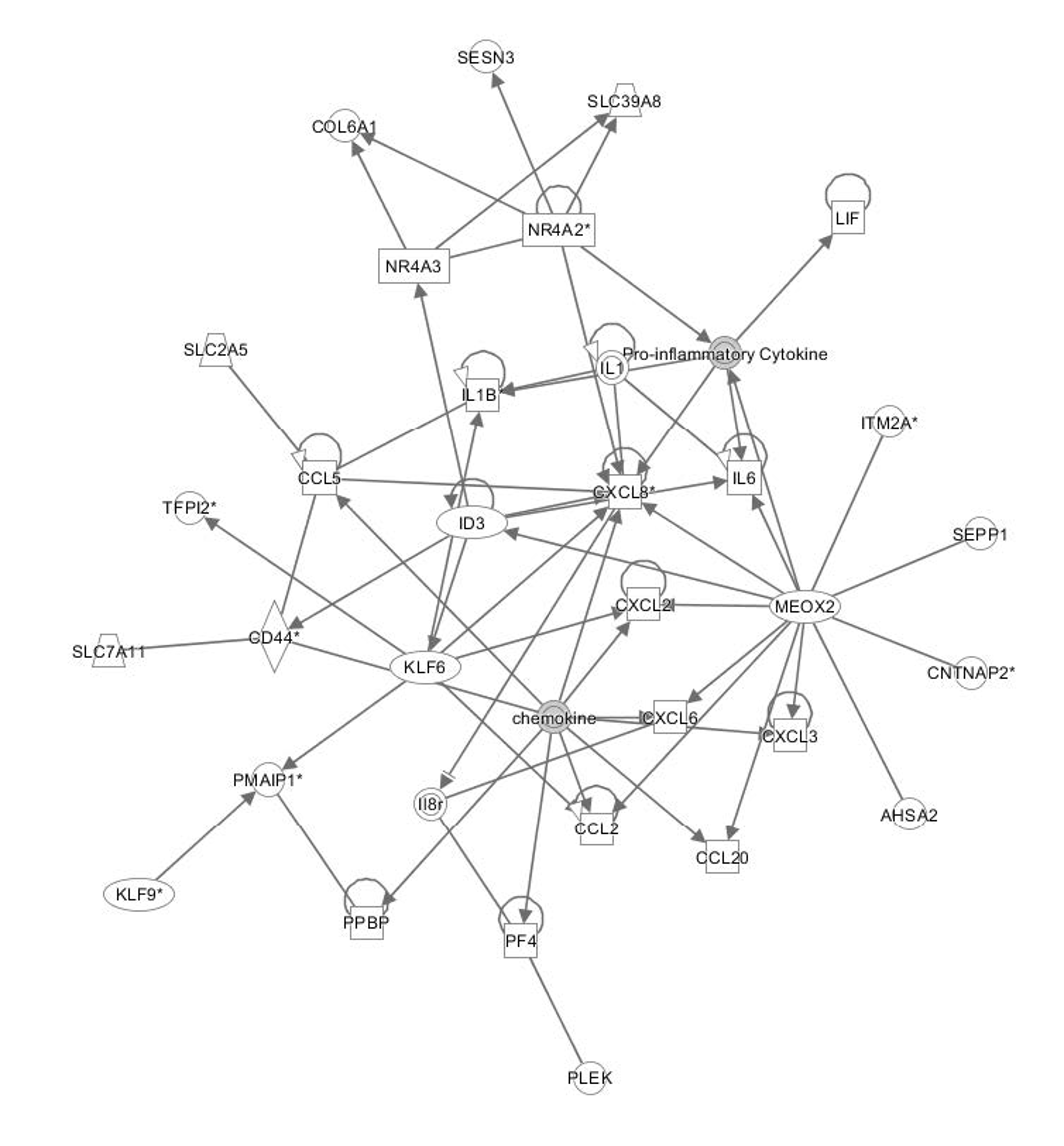

Supplement: S8 Fig — The plot is representative of cell-to-cell signaling and interaction, cellular movement, and immune cell trafficking networks. (TIFF) [file pone.0172430.s008.tiff]

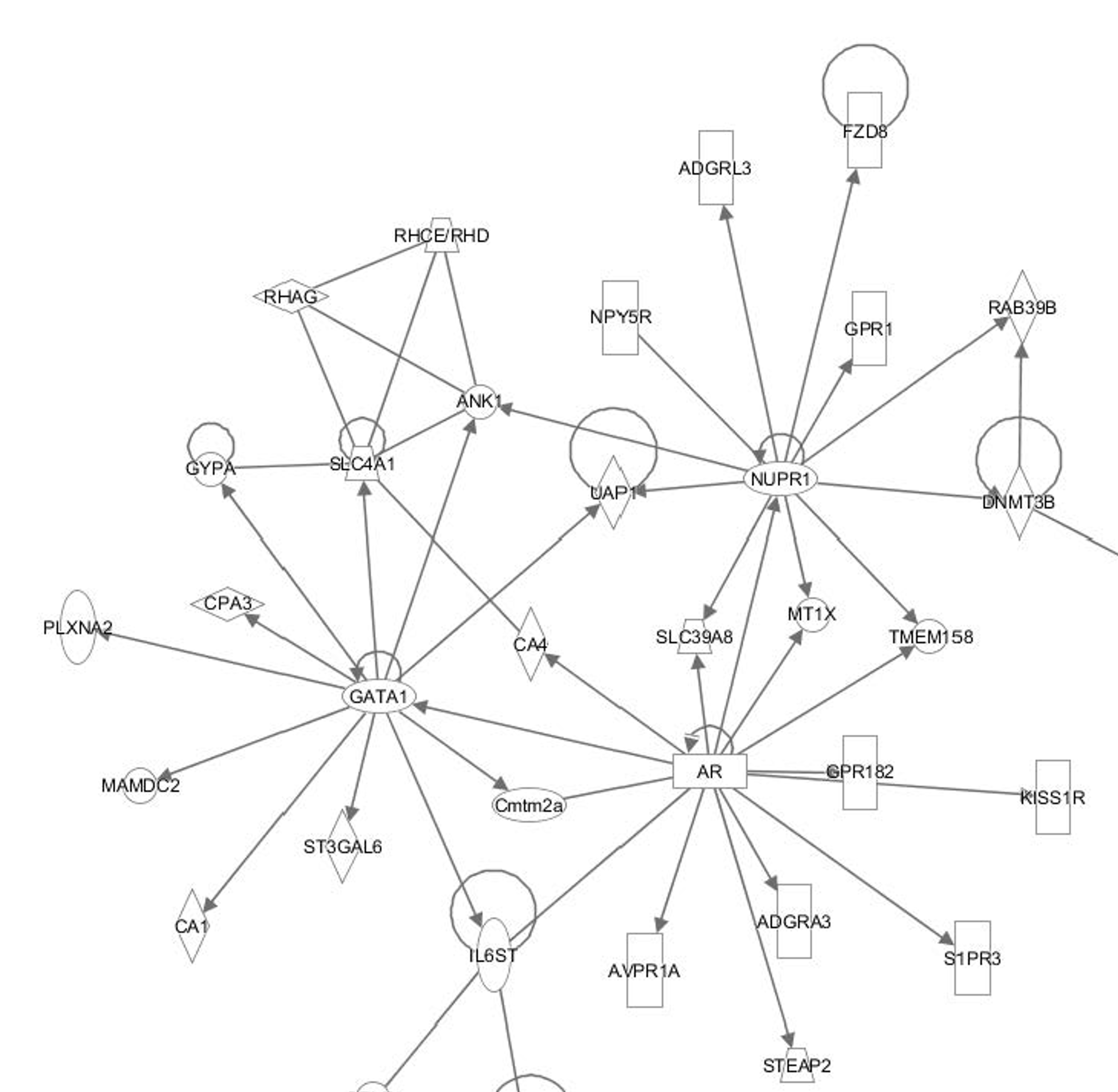

Supplement: S9 Fig — The plot is representative of cardiovascular system development and function, cellular development and haematological system development and function networks. (TIFF) [file pone.0172430.s009.tiff]
