# Supplementary material for: Mesenchymal stromal cells (MSCs) induce ex vivo proliferation and erythroid commitment of cord blood haematopoietic stem cells (CB-CD34+ cells)
Source: PLoS One. 2017 Feb 23;12(2):e0172430. doi: 10.1371/journal.pone.0172430 (PMC5322933; doi:10.1371/journal.pone.0172430)
Supplement: S1 Table — The table summarise the results about the assessment of the ex vivo expansion of CB-CD34+ cells grown in presence of MSCs. Briefly, 5×10^5^ CB-CD34+ cells were cultured alone or in presence of a layer of MSCs for 10 days; the fold increase in total cell number was calculated from the original CD34+ cells seeded at day 0. Absolute number of CB-CD34+ cells was measured by flow cytometry. Values derive as median of 5 replicates. Abbreviation: CB-alone: number of CB-CD34+ cells after 10 days of single culture; SN-fraction: CB-CD34+ cells in the supernatant (SN-fraction) of the co-cultures with MSCs; AD-fraction: CB-CD34+ cells grown directly in contact with MSCs layer; Total SN+AD: total number of CB-CD34+ cells after co-culture with MSCs. (DOC) [file pone.0172430.s010.doc]

| **Replicate** | **Fold Increase** | | |
| --- | --- | --- | --- |
|  | **Total** | **SN-fraction** | **AD-fraction** |
| 1 | 19.70 | 15.74 | 3.96 |
| 2 | 9.68 | 5.42 | 4.26 |
| 3 | 14.68 | 13.20 | 1.47 |
| 4 | 7.72 | 6.39 | 1.33 |
| 5 | 29.17 | 22.49 | 6.68 |
| **median** | 14.68 | 13.20 | 3.96 |
| **p-value**  **(vs CB-alone)** | <0.01 | <0.01 | ns |
