# Supplementary material for: Mesenchymal stromal cells (MSCs) induce ex vivo proliferation and erythroid commitment of cord blood haematopoietic stem cells (CB-CD34+ cells)
Source: PLoS One. 2017 Feb 23;12(2):e0172430. doi: 10.1371/journal.pone.0172430 (PMC5322933; doi:10.1371/journal.pone.0172430)
Supplement: S3 Table — The table reports the networks in which the differentially expressed genes (in bold characters) are involved. Columns list the Molecules in Network, the Score, the Focus-molecules and the direct related Top Diseases and Functions Molecules. (DOCX) [file pone.0172430.s012.docx]

| **Molecules in network** | **Score** | **Focus Molecules** | **Top Diseases and Functions** |
| --- | --- | --- | --- |
| **ACSBG1**, **AK7**, AK9, **ANPEP**, **ARHGEF2**, ARHGEF10, ARHGEF17, ARHGEF19, **BBS12**, C11orf31, **CCNA1**, CCT2, **DGKE***, DHRS7, **ETV5***, FXYD5, **GSTO2**, **JCHAIN**, KCNAB3, KRT23, **LAMP2**, **MSM01**, MVB12B, NCAM2, **NEDD4L**, PRRG1, RPP25L, SEZ6L2, **SLC18A2**, TMEM55A, **TPP2**, UBC, UBE38, ZADH2, ZC3HAV1L | 31 | 15 | Cell-to-Cell Signaling and Interaction, Connective Tissue disorders, Developmental Disorder |
| ADD3, **ARL4A**, CALCRL, **CKAP4***, **CR1L**, **EPRS**, FZD5, GPR183, Histone h3, **HIVEP3**, **HOXA9**, **HOXA10**, **ID1**, IRX3, KMT2A, LAT2, LCK, LGR4, **MARS**, **mir-196**, miR-196a-5p (and other miRNA w/seed AGGUAGU), OPN1LW, OPN1SW, **PALLD***, PTPRCAP, RSPO1, RSPO2, SBF1, SLC7A6, **SOS1**, **STAR**, WNT10B, ZNF22, **ZNF148**, **ZNRF3** | 30 | 15 | Cellular Growth and Proliferation, Hematological System Development and Function, Hematopoiesis |
| BCL2L12, **CAMTA1**, CARKD, **CCDC71L**, CDH15, **EVA1B***, FXYD7, **G2E3**, GHITM, HSPA13, IGLC1, IRAK2, ITPRIPL1, **METTL4**, MYDGF, NFXL1, NME3, PELI3, **RC3H2**, **RILPL1**, SGTA, **SLC16A9**, **SLC30A1**, SMAGP, SYT11, **TBC1D31**, TMC6, TMUB2, **TUBE1**, TWSG1, UBC, WBP1L, **WDR89**, **ZC3H12C**, **ZNF709** | 28 | 14 | Cardiovascular Disease, Developmental Disorder, Digestive System Development and Function |
| **ACVR1**, C6orf89, **CLCN3**, **DEPDC1**, ELFN1, ELFN2, **ENAH***, ESR1, FAM177A1, FAM35A, FBXO6, GINM1, **GPATCH2**, **GPR155**, HYDIN, IGSF3, **KIF18A**, LOC391322, LRRC58, miR-124-3p (and other miRNA w/seed AAGGCAC), NANOG, PLBD2, **PLXNA1**, PPP1CA, PPP1R27, **PTGFRN**, **RAB6B**, **RAPGEF5**, SLC44A2, TMEM132D, TTC17, TTC7A, UBC, **ZCCHC24**, **ZNF462*** | 26 | 13 | Developmental Disorder, Gastrointestinal Disease, Hereditary Disorder |
| **AIM2**, **ANKRD50**, APP, **ASPM**, **BMP2K**, C19orf12, C1orf105, C1QL1, C3orf33, C5orf15, CTIF, DENND1C, DTD2, ELAVL1, **KAZN**, MCTP2, MPV17L, MTFMT, NIM1K, **NMU**, NOTCH3, **PCGF2**, RALBP1, **SCML1**, SLC48A1, SMARCA4, **SYCP21**, TBC1D20, **THAP10**, **TMEM56**, **TMEM108**, TMEM127, TP53, **VASH2***, WDR45B | 24 | 13 | Cell Cycle, Cell Death and Survival, Cellular Compromise |
