# Supplementary material for: Mesenchymal stromal cells (MSCs) induce ex vivo proliferation and erythroid commitment of cord blood haematopoietic stem cells (CB-CD34+ cells)
Source: PLoS One. 2017 Feb 23;12(2):e0172430. doi: 10.1371/journal.pone.0172430 (PMC5322933; doi:10.1371/journal.pone.0172430)
Supplement: S5 Table — The table reports the networks in which the differential expressed genes (in bold characters) are involved. Columns list the Molecules in Network, the Score, the Focus-molecules and the direct related Top Diseases and Functions Molecules. (DOCX) [file pone.0172430.s014.docx]

| **Molecules in network** | **Score** | **Focus Molecules** | **Top Diseases and Functions** |
| --- | --- | --- | --- |
| **AHSA1**, **CCL2**, **CCL5**, **CCL20**, **CD44***, chemokine, **CNTNAP2***, **COL6A1**, **CXCL2**, **CXCL3**, **CXCL6**, **CXCL8***, **ID3**, IL1, **IL6**, **IL1B***, Il8r, **ITM2A***, **KLF6**, **KLF9***, **LIF**, **MEOX2**, **NR4A2***, **NR4A3**, **PF4**, **PLEK**, **PMAIP1***, **PPBP**, Pro-inflammatory Cytokine, **SEPP1**, **SESN3**, **SLC2A5**, **SLC39A8**, **SLC7A11**, **TFPI2*** | 49 | 31 | Cell-to-Cell Signaling and Interaction, Cellular Movement, Immune Cell Trafficking |
| Alcohol group acceptor phosphotransferase, **AURKA***, **AURKB**, **BIRC5***, **BUB1**, C/ebp, **CDC20**, **CDCA2**, **CDCA8**, **CDK1***, **CENPF***, **DAPK1**, **DLGAP5**, ERK, ERK1/2, **HMMR***, **ITPRIP**, **KIFF11**, **KIF15**, **KIF18A**, **KIF2C**, **KRT33A**, **MAD2L1***, **MAP2K6**, **MAP3K8**, **MFAP4**, **NCAPG**, **NCAPH**, **NEK2**, **NSFL1C**, **PPP1R13L**, **RMI2**, **TPX2**, **TRIB1**, **TTK** | 48 | 31 | Cell Cycle, Cellular Assembly and Organization, DNA Replication, Recombination, and Repair |
| 14-3-3, **ALDOC**, Alpha tubulin, **ARHGAP15**, **ARHGEF28**, **BCLAF1**, **CPE**, **CST7**, **CTSC***, **EIF3B***, **ELANE**, **EMP1**, **FUS**, **HAS3**, **HMGA1**, **HNRNPA1**, **HNRNPD**, **HNRNPL**, **ICAM1***, **IL7R**, Jnk, **LBP**, **LSAMP***, **MME**, **MYB**, **POU2F2***, Ras, **RNASE2**, **RPS23**, **RRAD***, **SCAF4**, **SNCA**, Tlr, **TLR2**, **TP63** | 47 | 30 | Cellular Assembly and Organization, Cellular Compromise, Connective Tissue Disorders |
| **ADAM19**, **ARHGAP31**, **ARHGDIB***, **BIRC3**, **CADM1***, **CHI3L1***, **DEPDC1***, **DOCK2**, **EPB41L3**, ETS, **HIST1H2AC**, Histone h3, **KCNMA1**, **LMO2**, LRP, **LRPAP1**, **MARCH3**, **MT1G**, **MTFR2**, **NCF4**, **PHF19***, Rac, **RAC2**, **SERPINE1***, Smad2/3, **TBX3**, **TGFBR1**, **TNFAIP3***, **TRIM23**, **UBE2D3**, Ubiquitin, **UFM1**, **UHRF1**, **WWC1**, **ZC3H12A** | 45 | 29 | Cellular Movement, Cellular Development, Cellular Growth and Proliferation |
| **ANK3***, **B2M**, BCR (complex), **BUB1B**, **CASC5**, CD3, **CENPE**, **E2F7**, **E2F8**, **HBB***, **HBD**, haemoglobin, IgG, **KIF4A**, **LAPTM5***, **MAFF***, **MBP***, **MELK**, MHC, MHC Class I (family), MHC Class II (complex), **NDC80**, **NFE2L3***, **NQO1**, **NUF2**, **PDPN***, **PRC1**, **PRSS23***, **PTPRC**, **RACGAP1**, **SCN2A**, **SHCBP1**, **SPC25**, TCR, **ZWINT** | 40 | 27 | Cell Cycle, Cellular Assembly and Organization, Hepatic System Development and Function |
| Ap1, **ARF1**, **CEP126**, Ck2, Collagen type VI, Creb, **DNER**, **ETS2**, **FHL1***, GPIIB-IIIA, **HES1***, **ITGA2B**, **ITGB3**, **JMJD1C**, **LAMA4**, **LMNB1**, **MT2A**, **MYO10**, **NAMPT***, NFkB (complex), **NUMB**, **PER1***, **PID1**, **RBPJ**, **RGS2**, **RSPO3**, Rxr, Smad, **SPAG4**, **THBS1***, thymidine kinase, **TK1**, **TM4SF1***, **TOP2A***, **VEGFA*** | 38 | 26 | Cardiovascular System Development and Function, Embryonic Development, Organismal Development |
| **AMPD3**, APC (complex), **BRIP1**, **CCNA2***, **CCNB1***, **CCNB2**, Cdc2, **CDC45**, **CDT1**, **CH25H**, **CHI3L2**, **CKS2**, **CP**, **CTSB***, Cyclin A, Cyclin B, E2f, **EREG**, **ERO1L***, **ESM1**, Growth hormone, **IRAK3**, Mcm, **MCM5**, **MCM10**, **MPO***, **NFKBIA**, **NUSAP1***, **ORC6**, **PRR11**, Rb, **RGCC**, RPA, **TYMS*** | 37 | 26 | Cell Cycle, Cellular Assembly and Organization, DNA Replication, Recombination, and Repair |
| 26s Proteasome, Actin, **AIF1***, Alpha Actinin, **CALD1**, Calmodulin, **CENPW**, **CLIP1**, **CORI1A**, **DMD**, **ENC1***, **ENO2**, Erm, F actin, G-actin, **GAS7**, Hsp70, Hsp90, **INSIG1***, **LCP1**, **LIMCH1***, **MLPH**, **MOK**, **MYOZ2**, **NACA**, **NPC1**, **PLD1**, **RAB27B**, **RAB3B**, **S100P**, **SGCG**, **SYTL4**, tubulin (complex), **TXNL1**, **UBE2C** | 35 | 25 | Cellular Assembly and Organization, Cellular Function and Maintenance, Hair and Skin Development and Function |
| **C3**, c-Src, **CA12***, Cbp/p300, **CD53**, **CD82**, **CDCP1***, **CFB**, **CSF2RB**, Cytokeratin, **DPT***, **DST**, **FANCI***, Fibrin, Gamma tubulin, **IFIT1**, **IGF1***, Integrin alpha 4 beta 1, **IRF1**, **KRT14**, **KRT16**, **KRT19**, **MND1**, N-cor, **PRELP**, **PSMB9**, **PTGS2**, **RAD51**, **RAD51AP1**, RNA polymerase II, **SIPA1L2**, STAT5a/b, **TGM2**, **USP32**, **XIST*** | 35 | 26 | Hematological Disease, Infectious Disease, Neurological Disease |
| 3’,5’-cyclin-nucleotide phosphodiesterase, **ADAMTS2**, **ADAMTS6***, **ADAMTS9**, **CRIP1**, **CTNNB1**, Ecm, **ECM2***, Fgf, **FGF5**, Fgfr, IFN Beta, Integrin alpha 3 beta 1, **IQGAP2**, Lfa-1, **LRRFIP1**, Mac1, Metalloprotease, Pde, PDE1/2/4, **PDE1A**, **PDE1C**, **PDE4B**, **PDE4D**, **PDE5A**, **PLAUR**, **PPAP2B***, **RCBTB2**, **SCD***, **SFRP1***, **SRPX2**, Tgf beta, **TNC**, **UGCG**, Vegf | 27 | 22 | Cell Signaling, DNA Replication, Recombination, and Repair, Nucleic Acid Metabolism |
| Akt, **CD84**, **EXOSC6**, Focal adhesion kinase, **GHR**, growth factor receptor, **HJURP**, Hsp27, **KIT**, **MK167**, **MPPED2**, **NTRK2**, P38 MAPK, p85 (pik3R), Pdgfr, **PDGRFB**, PI3K (complex), **PIK3AP1**, **PLAC8**, PLC gamma, **PLCL1**, PP1 protein complex group, PP1-C, **PPP1R3C**, Ppp2c, **RGS20**, **ROR2**, Shc, **SNAP25**, SRC (family), **SRSF3**, **STMN2**, **TRIP13**, tubulin (family), **VAMP8** | 26 | 20 | Cancer, Organismal Injury and Abnormalities, Endocrine System Disorders |
| **ACAT2**, **AK5***, **ATP1B1**, ATP6V0C, ATPase, BANF1, **BLM**, C6orf69, CARNS1, **CILP2**, **DCBLD2***, DQX1, **FAM6SC**, FBXO6, **GGH**, **GSPT1**, **HLA-B**, IGSF3, **KIF14***, **MEDAG**, MSR1, mt-Atp8, **NKG7**, **NRROS**, **PADI2***, PLBD2, **PNP**, RHOG, **RIMS3**, **SMIM3**, **SMOC2**, TRAF6, TTC17, UBC, YWHAZ | 24 | 19 | Connective Tissue Disorders, Dental Disease, Developmental Disorder |
| Cdkn3, **CDKN3***, **COL13A1**, **COL15A1**, **COL21A1**, COL22A1, COL28A1, COL6A5, COL6A6, collagen, Ctbp, CTNND1, **DUSP5**, DUSP18, Dusp21, **EYA1**, **IK2F1**, **IL18R1**, KRTAP10-7, **LIPG**, **MEST**, **MS4A3**, **NANOS1**, **PNLIPRP3**, PPARD, PTPase, PTPDC1, Ptpn14, **SULF2***, **TCEA3**, **THAP6**, **TMEM119**, triacylglycerol lipase, UBC, **UCP2** | 22 | 18 | Connective Tissue Disorders, Dermatological Diseases and Condition, Organismal Injury and Abnormalities |
| **BDKRB1**, **CALCRL**, Casein, **CMKLR1**, **COL7A1**, Collagen type I, Collagen type II, Collagen type III, Collagen type IV, Collagen type IX, Collagen type VII, Collagen(s), **CPM**, **CTSG**, **ELN**, **FBLN5**, **FGF7**, Fibrinogen, Gpcr, Igfbp, Integrin, **ITGA11**, JUN/JUNB/JUND, Laminin, LDL, Mmp, **MMP1**, **MMP3**, **MMP16**, **MMP28**, **PTGER2**, secreted MMP, Tenascin, **TGIF1**, **TIMP3** | 20 | 17 | Connective Tissue Disorders, Cardiovascular Disease, Post-translational Modification |
| ACTR5, ACTR8, **ASPM**, **CDCA7**, CLASP1, DAP, DGCR14, **EHBP1**, EHD2, EHD3, FAHD1, **HMGCS1***, HMGCS2, **IL32**, **INO80C**, LPIN2, LPPR3, LPPR4, **LRGI1**, **MT1X**, NQO2, OSBPL9, **OSBPL10**, **PHLDB2**, **PLXDC2**, **PPAP2A***, **PPAP2B***, PPAP2C, PREX1, **SAMSN1**, **TMF1***, **TTC14**, UBC, **UNC5B***, USP6NL | 20 | 17 | Carbohydrate Metabolism, Lipid Metabolism, Small Molecules Biochemistry |
| **ADIRF**, AP3B2, ARR3, BEGAIN, **BEX1**, CAPNS1, CYTH1, DBT, ELOVL1, **ELOVL2**, **ELOVL6***, **EPG5**, FIS1, **FRAS1**, **GDAP1**, **GINS1**, GINS3, GINS4, GTF2IRD1, MC2R, **MT1F***, NOP2, PLK4, PTAFR, **QSOX1**, RHO, **RNASE6**, RPS6KA6, **SLCA12**, **SLC38A1***, **TTLL5**, UBC, **ZFAND6**, **ZNF287**, ZNF496 | 19 | 16 | Lipid Metabolism, Small Molecules Biochemistry, Hereditary Disorder |
| AK7, AK9, **ANLN, BHLHE40*, BTG1, CLDN11, CMPK2, DPYSL3, DTL*, EPHA3,** FAIM, **KIAA0101,** NTSC, NT5C1B, **NT5E*,** NUBP1, **OIP5,** PARG, **PGAM2,** PGAM4, **PLCB4**, PLCD3, PLCH1, PLCH2, PLD4, RPL39, **RPL22L1,** RPL39L, RPL3L, RTFDC1, **SPRY2, SYNE1,** TRIM51, UBC, ZC3HAV1L | 19 | 16 | Nucleic Acids Metabolism, Small Molecules Biochemistry, DNA Replication, Recombination, and Repair |
| AAMDC, ADI1, ANKRD39, APP, BAG3, C21orf59, C4orf22, CCDC88C, **CCNL1,** CRACR2B, **DEPTOR,** DNAJB3, DNAJB14, DNAJC4, **DNAJC12,** DNAJC19, DPCD, **EVI2A, FAM213A, FAM64A, FAM84A,** GALE, **GSAP,** HAGH, INIP, **NDP,** PAIP2, **PSAT1*,** RASL11B, **RGS18, SH3RF2, SPSB2, STRIP2*, SULF1*, ZMYM5** | 17 | 16 | Cardiovascular System Development and Function, Cellular function and Maintenance, Cellular Growth and Proliferation |
| **C16orf54, CKAP2L,** CYHR1, **DEPDC1B,** DHX33, DUSP2, GSTM2, **KIAA1551,** LIMK2, LMAN2, MAP3K6, MAPK4, MAPK9, **MOXD1*, MT1E,** NEU3, Pak2, POMK, PRKX, **RASEF,** SH3BP5, **SHISA2, SLC14A1*, SLC38A5,** SLK, **SMC03,** SSU72, **STEAP1, TMED5, TPBG,** TUSC2, UBC, XPNPEP1, ZBTB25, **ZNF93** | 17 | 15 | Developmental Disorder, Hematological Disease, Hereditary Disorder |
| ABCG1, ARNT2, **CELF2*,** CPXM2, **CSRNP1,** DIRA53, DUSP11, ELAVL1, **FAM13B, FAM84B,** FKBP9, **FLRT3*,** GALNT4, GIGYF1, **HACD2,** HOXC11, MARVELD2, **MIDN,** MTMR4, MTMR9, NFATC1, PDCD1, **PPIC,** PPIL3, PPTC7, **SEPT11*, SIGLEC15, SLC2A3*,** SLC39A10, STAT3, **TCF7L2,** **TMTC4,** Tnfsf9, **TRIM14,** ZDHHC14 | 17 | 15 | Immunological Disease, Inflammatory Disease, Inflammatory Response |
| ANGPTL4, **ARHGAP28,** CAMK1, CNTN1, DNAJB5, DNAJB8, DNAJC1, **ELOVL6*, GIMAP2, GMFG,** GPM6A, GSTO1, HDAC4, HOXC9, HSPB1, **LOC344887, LPXN, MCTP2*,** MEF2, MPHOSPH6, **MT1H,** MXD1, **NAP1L3,** NR2C1, NRIP3, PCSK9, **PHLDA1*,** PHLDB1, **SCRG1,** SERPINB2, **SLC43A3*,** SMARCA4, **SPOCD1*,** SS18L1, TSPANI3 | 15 | 14 | Lipid Metabolism, Nucleic Acids Metabolism, Small Molecules Biochemistry |
| **ATP10A,** BRCA1, **CD24*, CENPH,** CENPI, CENPK, CENPL, **CENPN,** CENPO, CENPP, CENPQ, **CENPU,** CRYZL1, DCLRE1B, **EPHX1,** FMO5, **GINS2,** GINS3, HNF4A, **HSD11B1,** ITIH3, ITIH4, **ITIH5*, MATN3,** MATN4, **MMAA,** MUT, PERP, **PFKFB4,** RPRD18, **SLC38A4,** SPATC1L, TAPBPL, TCF4, **THAP2** | 15 | 14 | Lymphoid Tissue Structure and Development, Metabolic Disease, Developmental Disorder |
| ADGRA3, ADGRL3, ANK1, AR, AVPR1A, CA1, CA4, **CLC,** Cmtm2a, **CPA3,** CRLF1, **DNMT3B, FZD8,** GATA1, GPR1, GPR182, GYPA, **HELLS,** IL6ST, KISS1R, **MAMDC2, MT1X,** NPTSR, NUPR1, **PLXNA2, RAB39B, RHAG,** RHCE/RHD, S1PR3, **SLC39A8,** SLC4A1, **ST3GAL6, STEAP2, TMEM158,** UAP1 | 15 | 14 | Cardiovascular System Development and Function, Cellular Development, Hematological System Development and Function |
